# Short-chain fatty acids induced lung tumor cell death and increased peripheral blood CD4+ T cells in NSCLC and control patients ex vivo

**DOI:** 10.3389/fimmu.2024.1328263

**Published:** 2024-04-08

**Authors:** Carolin D. Thome, Patrick Tausche, Katja Hohenberger, Zuqin Yang, Susanne Krammer, Denis I. Trufa, Horia Sirbu, Joachim Schmidt, Susetta Finotto

**Affiliations:** ^1^ Department of Molecular Pneumology, University Medical School Hospital Erlangen (UKER) Friedrich-Alexander-University (FAU), Erlangen-Nürnberg, Germany; ^2^ Department of Thoracic Surgery, University Medical School Hospital Erlangen (UKER), Friedrich-Alexander University of Erlangen-Nürnberg (FAU), Erlangen, Germany; ^3^ Bavarian Cancer Research Center (BZKF), Erlangen, Germany; ^4^ Comprehensive Cancer Center Erlangen-EMN (CCC ER-EMN), Erlangen, Germany; ^5^ Department of Anesthesiology, University Medical School Hospital Erlangen (UKER), Friedrich-Alexander University of Erlangen-Nürnberg (FAU), Erlangen, Germany

**Keywords:** lung cancer, NSCLC, short chain fatty acids (SCFA), sodium butyrate, A549, T cells, IFN-γ-receptor, glucose

## Abstract

**Background:**

Despite therapy advances, one of the leading causes of cancer deaths still remains lung cancer. To improve current treatments or prevent non-small cell lung cancer (NSCLC), the role of the nutrition in cancer onset and progression needs to be understood in more detail. While in colorectal cancer, the influence of local microbiota derived SCFAs have been well investigated, the influence of SCFA on lung cancer cells via peripheral blood immune system should be investigated more deeply. In this respect, nutrients absorbed via the gut might affect the tumor microenvironment (TME) and thus play an important role in tumor cell growth.

**Objective:**

This study focuses on the impact of the short-chain fatty acid (SCFA) Sodium Butyrate (SB), on lung cancer cell survival. We previously described a pro-tumoral role of glucose on A549 lung adenocarcinoma cell line. In this study, we wanted to know if SB would counteract the effect of glucose and thus cultured A549 and H520 *in vitro* with and without SB in the presence or absence of glucose and investigated how the treatment with SB affects the survival of lung cancer cells and its influence on immune cells fighting against lung cancer.

**Methods:**

In this study, we performed cell culture experiments with A549, H520 and NSCLC-patient-derived epithelial cells under different SB levels. To investigate the influence on the immune system, we performed *in vitro* culture of peripheral mononuclear blood cells (PBMC) from control, smoker and lung cancer patients with increasing SB concentrations.

**Results:**

To investigate the effect of SB on lung tumor cells, we first analyzed the effect of 6 different concentrations of SB on A549 cells at 48 and 72 hours cell culture. Here we found that, SB treatment reduced lung cancer cell survival in a concentration dependent manner. We next focused our deeper analysis on the two concentrations, which caused the maximal reduction in cell survival. Here, we observed that SB led to cell cycle arrest and induced early apoptosis in A549 lung cancer cells. The expression of cell cycle regulatory proteins and A549 lung cancer stem cell markers (CD90) was induced. Additionally, this study explored the role of interferon-gamma (IFN-γ) and its receptor (IFN-γ-R1) in combination with SB treatment, revealing that, although IFN-γ-R1 expression was increased, IFN-γ did not affect the efficacy of SB in reducing tumor cell viability. Furthermore, we examined the effects of SB on immune cells, specifically CD8+ T cells and natural killer (NK) cells from healthy individuals, smokers, and NSCLC patients. SB treatment resulted in a decreased production of IFN-γ and granzyme B in CD8+ T cells and NK cells. Moreover, SB induced IFN-γ-R1 in NK cells and CD4+ T cells in the absence of glucose both in PBMCs from controls and NSCLC subjects.

**Conclusion:**

Overall, this study highlights the potential of SB in inhibiting lung cancer cell growth, triggering apoptosis, inducing cell cycle arrest, and modulating immune responses by activating peripheral blood CD4+ T cells while selectively inducing IFN-γ-R1 in NK cells in peripheral blood and inhibiting peripheral blood CD8+ T cells and NK cells. Thus, understanding the mechanisms of action of SB in the TME and its influence on the immune system provide valuable insights of potentially considering SB as a candidate for adjunctive therapies in NSCLC.

## 1 Introduction

Lung cancer is one of the leading causes of cancer-related deaths, gender-independent, worldwide. It is a complex disease that arises from genome damage in healthy lung cells, which then results in the uncontrolled growth of abnormal cancerous cells in the lung tissue. Around 85% of lung cancer cases are attributed to non-small cell lung cancer (NSCLC). The primary risk factor for lung cancer is tobacco smoking, 81% of lung cancer deaths are directly caused by it, but other factors such as exposure to air pollution and genetic predisposition also play a role (1).

In recent years, significant progress has been made in understanding the molecular and genetic mechanisms underlying lung cancer development, which has led to the development of targeted therapies and immunotherapies. However, despite these advances, the prognosis for lung cancer remains poor, particularly for patients diagnosed at advanced stages of the disease (2, 3).

Nutrients in the TME play a critical role in tumor cell growth. One of the key nutrients is glucose that is essential for the survival and proliferation of the tumor, as tumor cells mainly depend on aerobic glycolysis, an effect called Warburg effect (4). To further investigate, how the diet influences tumor growth via the gut-lung-axis, a bidirectional communication between the gut and the lungs, involving immune modulating components like microbial components, metabolites and signaling molecules, which influence the health of both organs (5), we focused on short-chain fatty acids (SCFA), which are produced in the gut via fermentation of non-digestible dietary fibers by commensal probiotic bacteria. Most typical examples are acetate (C2), propionate (C3) and butyrate (C4; sodium butyrate, SB), which was the primary focus of our study (6). SB is one of the main energy sources in the colonocytes and has been shown to inhibit the cell growth of the colorectal cancer cell line HT29 effectively (7). As shown previously, the composition and function of gut microbiota was significantly changed in patients with lung cancer compared to healthy controls. This is associated with fewer probiotics, increased facultative pathogenic bacterial populations, and therefore decreased circulation of SCFAs (8). SB has been shown to have anti-cancer effects in colorectal cancer (CRC), like suppressing the proliferation of cancer cells by inhibiting histone deacetylase (HDAC) activity. This increases histone acetylation, which relaxes the chromatin structure and allows the expression of tumor suppressor genes (6). As described previously, cancer cells rely more on glycolysis than on fatty acid oxidation (Warburg effect), which is a reason why SB is not degraded via oxidative phosphorylation. Therefore, SB accumulates more in CRC cells than non-cancerous epithelial cells and leads to inhibition of HDAC and cellular proliferation only in cancerous cells, but not in normal colonic epithelium, which is the Butyrate-Paradox (9).

As well as for the epithelial cells previous studies reported increased proinflammatory cytokine production, like IFN-γ, granzyme B and perforin, of CD8+ T cells due to SB treatment (10). Additionally, SCFA have also shown promising effects on T cell differentiation with promoting either a proinflammatory subset to produce IL-17, IFN-γ or IL-10 depending on the predominant cytokine milieu. Thus, the effect of SCFA may be modulated by active immune responses (11). This effect may be promising in the fight of T cells against cancer cells.

Since SB has been shown to be beneficial in CRC, and additionally other types of cancer, such as for example prostate cancer and neuroblastoma cells (12, 13), NSCLC may be affected by the same mechanisms. Therefore, we investigated the role of extracellular SB concentration in the lung TME and its role in controlling tumor growth *in vitro*.

## 2 Materials and methods

### 2.1 Lung carcinoma cell culture experiments with different SB concentrations

The A549 lung adenocarcinoma (LUAD) cell line and H520 squamous lung cancer cell lines were purchased from the ATCC bank. For cell culture experiments 5x10^5^ A549 or H520 cells per well (**Supplementary Table S1**) were incubated at 37°C, 5% CO2 and 95% relative humidity in 6-well plates for 48h or 72h in 2ml RPMI 1640 medium containing 0 (Gibco™, Cat#11879020) or 200 (Anprotec Cat#AC-LM0060) mg/dl glucose. Furthermore, 0, 1mM or 5mM concentrations of sodium butyrate (Sigma, Cat#B5887-1G), and 0 or 25ng/ml rhIFN-γ (Immunotools, Cat#11343534) were added to the culture medium, listed in **Supplementary Table S2**.

### 2.2 Human subjects and study population

The underlying study was performed at Friedrich-Alexander-University, Erlangen (Germany) with the approval of the University of Erlangen’s ethics review board (Re-No: 22-64B; DRKS-ID: DRKS00029641). 9 NSCLC patients provided their consent and underwent surgery. Patient confidentiality was maintained throughout the study.

Peripheral blood mononuclear cells (PBMCs) were isolated from collected blood samples and cultured as described below.

Bronchoalveolar lavage fluid (BALF) from the healthy lung and the tumorous site were collected. Moreover, tissue samples of the surgically removed lung material were collected and categorized into the tumoral area (TU, solid tumor tissue), a peritumoral area (PT, 2cm around tumor), and tumor-free control area (CTR, at least 5cm away from the solid tumor).

Lung cancer diagnosis and histological subtype classification were confirmed by the Institute of Pathology at the University Hospital Erlangen. The TNM Staging followed the International Association for the Study of Lung Cancer (IASLC) proposals for revision of the TNM Stage Groupings in the Forthcoming (Eight) (TNM8) Edition of the TNM Classification for Lung Cancer in 2016. Relevant data were provided by the Department of Thoracic Surgery and the Institute of Pathology at the University Hospital in Erlangen and reported in **Supplementary Table S3**.

### 2.3 Human cell isolation and culture

The human epithelial cells from lung cancer patients were cultured on collagen precoated wells. For each well (48 well plate) 20µl 0,2% Collagen R (SERVA, Cat#47254), 2.5µl of 0.9% NaCl and 2.5µl of 0.17 M NaOH were spread over the well surface, incubated for at least 1 hour and washed 3 times with cold PBS.

The patient derived BALF was centrifuged at 1500rpm, 4°C for 5 min. Afterwards the supernatant (SN) was discarded, the cell pellet resuspended, and the cells cultured as described below.

For the lung cell isolation, the tissue samples were cut into small pieces, followed by collagenase digestion using 30 U/ml Collagenase from Clostridium histolyticum (Sigma-Aldrich, Cat# C98991-500MG) and 1,5 mg DNase (10 mg/ml; Roche Diagnostics GmbH: DNase I; Cat#10104159001) diluted in 10 ml RPMI 1640 medium (Gibco™, Cat#11879020) overnight at 37°C in a shaker. The digested lung was filtered through a 100µm cell strainer (Greiner Bio-One, Cat#542000). The cells suspension was centrifuged (10 min, 1500 rpm, 4°C) and the supernatant removed. The cell pellet was resuspended in ACK-lysis buffer (0,15M NH4Cl, Carl Roth GmbH + Co. KG, Cat# P726.2; 0,01M KHCO3, Carl Roth GmbH + Co. KG, Cat#P748.1; 100M Na2EDTA, GERBU Biotechnik GmbH, Cat# 1034.1000; dissolved and sterile filtered in deionized H2O; pH=7.2-7.4) to be incubated for 2 min, followed by centrifugation as described above. After supernatant removal cells were resuspended in PBS (anprotec, Cat# AC-BS-0002), following a slow pouring out of the 10ml pipette to remove residual fat. After a next centrifugation step, cells were resuspended in 10 ml PBS. Cell numbers and viability were determined by using Trypan-blue staining in a Neubauer counting chamber.

In preparation for subsequent cell culture experiments 3-5x10^5^ isolated lung cells were seeded into 48 well plates, precoated with collagen, in 500µl Pneumacult medium (1:1 Pneumacult+: Pneumacult++) on the base of Pneumacult Ex Plus Basal medium (Stemcells Technologies™ Cat#05041) with supplements as displayed in **Supplementary Table S4**. After an epithelial layer had formed, the cells were detached and transferred into bigger wells. This was repeated for about 2 weeks until the cell numbers were high enough to perform the experiments.

For the cell culture experiments 3 x10^5^ cells were incubated at 37°C, 5% CO2 and 95% relative humidity in 48-well plates for 48h or 72h in 427µl Pneumacult+ and 73µl Pneumacult++ medium (**Supplementary Table S4**). Furthermore, SB (Sigma, Cat#B5887-1G), 0, 1mM or 5mM was added to the culture medium.

### 2.4 PBMC isolation and culture

PBMC isolation was performed from freshly obtained EDTA blood using Leucosep Tubes (Greiner Bio-One, Cat#227290) according to the manufacturer´s protocol. For the separation step BioColl (Bio&Sell, Cat#BS.L6115) was used.

For cell culture, 5x10^5^ PBMC were incubated for 96h in 500µl RPMI 1640 medium containing 0 (GibcoTM, Cat#11879020) or 200 (anprotec Cat#AC-LM0060) mg/dl glucose. In some wells, the culture medium was supplemented with 1 or 5 mM SB. After 72h, cells were analyzed by flow cytometry. For the cytokine analysis of the T cells, the PBMC were treated at the beginning of the cell culture with 2.5 µg soluble anti-CD3 (BD Pharmingen Cat#555329) and 0.5µg anti-CD28 (BD Pharmingen, Cat#555752) (**Supplementary Table S2**). To inhibit the intracellular protein transport, cells were treated for 4h before harvesting with 1.5µl Golgi-stop (BD Pharmingen, Cat#51-2092KZ), 0.5µg PMA (Sigma Aldrich, Cat#524400) and 0,3nM Ionomycin solution (Sigma Aldrich, Cat#I3909-1ML) Protein expression of IFN-γ and granzyme B was measured by FACS.

### 2.5 Flow cytometric analysis of human cell lines, patient- derived epithelial cells and PBMC

To analyze surface antigen expression, 4x10^5^ cells were placed in a 96 well U-bottom plate and centrifuged at 2000 rpm for 1 minute at 4°C. After removing the supernatant, cells were resuspended and pre-incubated with anti-CD16/CD32 (1:100, BioLegend, Cat#422302) antibody for at least 5 minutes at 4°C in the dark to prevent non-specific binding of immunoglobulins to Fc receptors and centrifuged as above. After removing the antibody solution, cells were washed with 150 µl FACS-buffer (Lonza PBS EDTA (Cat#11665100) + 2% FCS (Sigma-Aldrich, Cat# S0615)) and centrifuged. Cells were resuspended and stained with a 50 µl surface antibody mix targeting specifics surface antigens for 20 minutes at 37°C in the dark. Following another wash step by addition of 150 µl FACS-buffer and centrifugation, the stained cell pellet was resuspended in 100 µl FACS-buffer and analyzed using FACS Canto II (BD Biosciences, Heidelberg).

To perform intracellular staining, cells were resuspended subsequently after the completed surface staining in 100 µl of FoxP3 Fixation/Permeabilization reagent (1 part FoxP3 Fixation/Permeabilization concentrate and 3 parts Fixation/Permeabilization Diluent, Thermo Fisher Scientific, Cat#00-5523-00) for 30 minutes at room temperature in the dark, then washed two times with 200 µl of permeabilization buffer (1 part Thermo Fisher Scientific Permeabilization Buffer (10X), Cat#00-5523-00, and 9 parts Millipore-H2O). 50 µl of the antibody mix diluted in permeabilization buffer was added, and cells were incubated for 30 minutes at room temperature in the dark for staining of intracellular epitopes. After two wash steps of adding 200 µl of permeabilization buffer and centrifugation, cells were resuspended in 100 µl FACS-buffer and analyzed using a flow cytometer (BD FACS Canto II, BD Biosciences, Heidelberg).

The applied antibodies are listed in **Supplementary Table S5**. Data sets were analyzed with Kaluza Flow Cytometry Software v2.1 (Beckman Coulter, Inc.).

### 2.6 FACS apoptosis assay

Apoptosis assay was performed with 1x10^5^ cells after 72h incubation by staining the cells with Annexin V (BD Pharmingen, Cat#550474), Propidium Iodide (PI) (BD Pharmingen, Cat#556463) and Annexin V-PI-Binding Buffer (BD Pharmingen, Cat#556454) following the manufacturers protocol. Flow cytometric analysis was performed with FACS Canto II, BD Biosciences, Heidelberg.

### 2.7 RNA isolation and quantitative real-time PCR

Total RNA was extracted from cell suspension samples using QIAzol Lysis^®^ Reagent (QIAGEN, Cat#79306) according to the manufacturer’s instructions. Of the resulting RNA 1µg was reverse transcribed into copy DNA (cDNA) using the Revert Aid™ First Strand cDNA Synthesis Kit (Thermo Fisher Scientific, Cat#K1622) according to the manufacturer’s instructions. The qPCR reaction mixture for each sample contained 15 ng of cDNA, 300 nM of transcript-specific forward and reverse primer iTaq Universal SYBR Green Supermix (Bio-Rad Laboratories, Cat# 1725124), in a total volume of 20 µl. The qPCR primers were obtained from Eurofins-MWG-Operon (Ebersberg, Germany). The primer sequences used for human qPCR analysis are shown in the **Supplementary Table S6**. Reactions were performed for 50 cycles, starting with initial activation for 2 minutes at 98°C, followed by cyclic denaturation for 5 minutes at 95°C, hybridization, and elongation for 10 minutes at 60°C. The CFX-96 Real-Time PCR Detection System (BIO-RAD, Munich, Germany) was used to perform the qPCR reactions, and data was analyzed using the CFX Manager Software (BIO-RAD). The relative expression level of specific transcripts was calculated using the relative quantification ΔCT method with reference to the internal standard ribosomal protein L30 (RPL30).

### 2.8 Statistical analysis

Statistical analyses were performed using GraphPad Prism 8 software to obtain significance levels (*P < 0.05; **P < 0.01; ***P < 0.001; ****P < 0.0001). Data, imported in column statistics, were analyzed for normal distribution with the Shapiro-Wilk test. One-way ANOVA and two-way ANOVA multiple comparison were used for normally distributed data. For non-normal distribution the Kruskal-Wallis test was used. Data are given as mean values ± SEM.

## 3 Results

### 3.1 SB treatment *in vitro*, leads to reduced lung cancer cell viability with or without glucose in the cell culture medium

**Figure 1:**
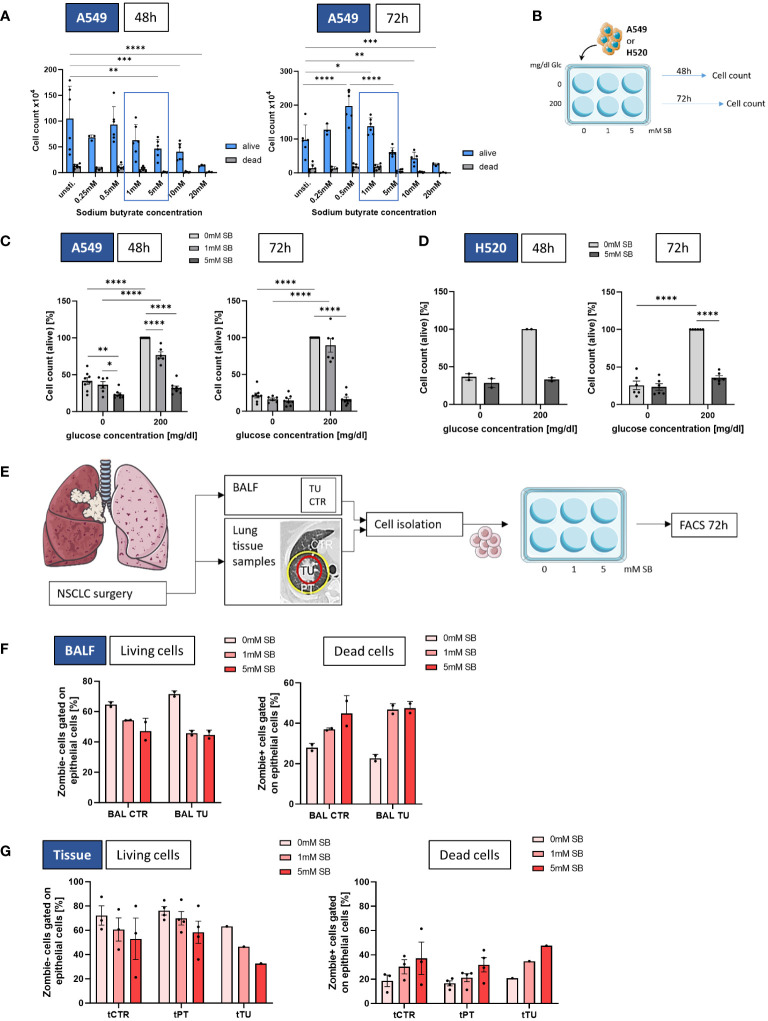
SB reduced cell viability in lung cancer cell lines A549 and H520, as well as in NSCLC-patient derived epithelial cells. **(A)** Cell count of living and dead A549 cultured with different SB concentrations (0-20mM SB) after 48h (left panel) and 72h (right panel); **(B)** Schematic illustration of the experimental design. 5x10^5^ A549 or H520 cells per well were incubated for 48 or 72h with 200mg/dl glucose or without and SB concentrations increased from 0mM over 1mM to 5mM SB; **(C)** Percentages of the cell count of living A549 cells stained with trypan blue solution after 48h or 72h of SB treatment 48h in relation to the control population (200mg/dl glucose, 0mM SB, (n=8); **(D)** Percentages of the cell count of living H520 cells stained with trypan blue solution after 48h (n=2) or 72h (n=6) of SB treatment in relation to the control population (200mg/dl glucose, 0mM SB),; **(E)** Schematic illustration of the experimental design of the human study. BALF- or tissue-derived cells were cultured in collagen coated wells for 72h with increasing SB concentrations from 0mM over 1mM to 5mM SB and analyzed by FACS; **(F)** Percentages of Zombie- (living) CD326+ epithelial cells or Zombie + (dead) CD326+ epithelial cells cultured from BALF in control regions and tumor regions with different SB concentrations analyzed by flow cytometry, (n^control^=2; n^tumor^=2); **(G)** Percentages of Zombie- (living) CD326+ epithelial cells or Zombie + (dead) CD326+ epithelial cells cultured from lung tissue samples in control, peritumoral and tumor regions with different SB concentrations analyzed by flow cytometry, (n^control^=3, n^peritumoral^=4, n^tumor^=1); (*P < 0.05; **P < 0.01; *** P<0.001; ****P < 0.0001). Two-way ANOVA test was used for figure **(A)**, one-way ANOVA test was used for figure (**C, D, F, G** left panel), Kruskal-Wallis test was used for figure (**G** right panel). All data are presented as mean values ± SEM.; Parts of the figure were drawn by using pictures from Servier Medical Art and is licensed under a Creative Commons Attribution 3.0 Unported License (https://creativecommons.org/licenses/by/3.0/).

To determine the most effective concentration of SB, A549 cells were cultured for 48 and 72h using varying concentrations from 0 to 20mM SB. Living and dead cells were quantified using trypan blue staining in a Neubauer counting chamber. The cell count decreased in a concentration dependent manner with increasing concentrations from 1 to 20mM SB compared to cells treated with 0mM SB (**Figure 1A**). Notably only 0.5mM SB showed higher counts of living cells than the 0mM SB treatment at 72h of cell culture. According to the concentration dependent response observed, we decided to proceed with 0, 1 and 5mM SB concentrations for subsequent experiments, (**Figure 1A**).

In order to understand the impact of SB within the lung cancer cell microenvironment, the LUAD cell line A549 or the squamous cell carcinoma H520 was cultured either without, with 1mM or with 5mM SB in the cell culture medium for 48h or 72h and each condition was investigated also without glucose (0mg/dl) or in the presence of glucose (200mg/dl) in the cell culture medium (**Figure 1B**). In A549 cells cultured in 200 mg/dl glucose, a significantly reduced cell count was observed with 5mM SB treated cells compared to untreated cells after 48h and 72h. However, the 1mM SB treatment significantly reduced the cell count only after 48h. Under glucose-deprived conditions, a decrease in cell count was evident after 48 hours in 5mM SB, as well as in comparison with 1 to 5mM SB (**Figure 1C**). The results in **Figure 1C** also show distinct growth restriction caused by glucose deprivation, which we described previously (14), resulting in a reduced cell count after 48 and 72h in the absence of SB stimulation. The addition of 5mM SB resulted in a further reduced average percentage of cell count in the absence of glucose. Similarly, in the presence of glucose the percentage of viable cells was reduced after 5mM SB, demonstrating a consistent effect of SB on tumor cell death after 48 and 72h, regardless of the presence or absence of glucose.

To evaluate the broader applicability of these findings beyond A549, we extended our examination of SB to H520 cell line (**Figure 1D**). The cell count percentages after 48h and 72h in the absence of glucose were generally low and significantly reduced after 72h in comparison to the 200mg/dl glucose and 0mM SB condition. In the 200mg/dl glucose condition the viable cells show a tendency of reduction upon 5mM SB treatment after 48 and were significantly reduced after 72h (**Figure 1D**).

To confirm the results of the experiments with the A549 and H520 in concurrent human samples, cells from BALF and lung tissue samples from NSCLC patients after surgery were cultured under epithelial cell supporting conditions and subjected to the same experiments as the A549 (**Figure 1E**). Because primary cells are more difficult to culture, a special cell culture medium was needed, and glucose was indispensable. Here we stained the dead cells with Zombie and analyzed this by FACS. For the BALF derived epithelial cells the living (Zombie-) viable cells tended to be reduced and the dead (Zombie+) cells tended to increase after SB treatment for 72h accordingly (**Figure 1F**). To see if there is some difference regarding the origin of the cells, we also investigated the cells grown from tissue samples, where we collected the control- (CTR), peritumoral- (PT) and the tumoral (TU) regions (**Figure 1E**). Based on the BEC results reported earlier, treatment with SB for 72 hours led to a decreasing trend but without significance in the percentages of living cells after 5mM SB treatment and a tendency to increase in the percentages of dead cells in a similar manner in all regions (**Figure 1G**).

### 3.2 SB induced IFN-γ-R1 surface expression and combined treatment of SB and rhIFN-γ led to reduced viable lung cancer cells

**Figure 2:**
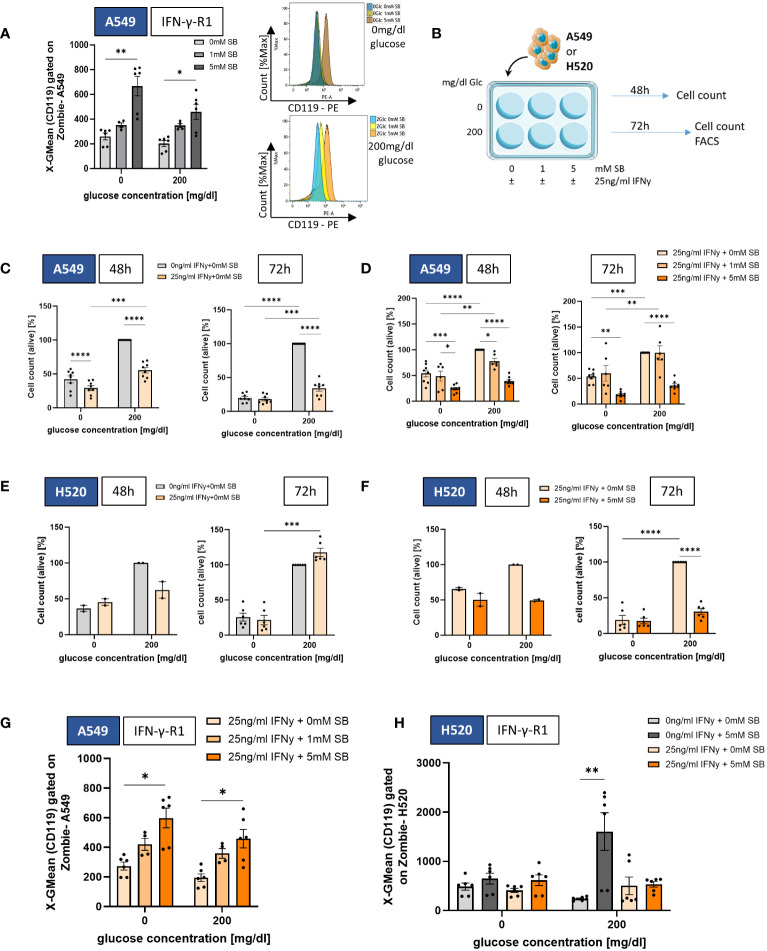
SB induced IFN-γ-R1 surface expression and reduced cell viability in combined treatment with rhIFN-γ. **(A)** Geometric mean of CD119 expression on A549 cell surface after 72h cell culture with 0, 1 and 5mM SB treatment and corresponding histograms without glucose (top) and with 200mg/dl glucose (bottom), analyzed by flow cytometry, (n=6); **(B)** Schematic illustration of the experimental design. 5x10^5^ A549 or H520 cells per well were incubated for 48h or 72h with 200mg/dl glucose or without and SB concentrations increased from 0mM over 1mM to 5mM SB with additionally 25ng/ml rhIFN-γ; **(C)** Percentages of the cell count of living A549 cells stained with trypan blue solution after 48h or 72h of rhIFN-γ treatment in relation to the norm population (200mg/dl glucose, 0ng/ml rhIFN-γ, 0mM SB), (n=8); **(D)** Percentages of the cell count of living A549 cells stained with trypan blue solution after 48h or 72h of SB and rhIFN-γ treatment in relation to the norm population (200mg/dl glucose, 25ng/ml rhIFN-γ 0mM SB), (n=8); **(E)** Percentages of the cell count of living H520 cells stained with trypan blue solution after 48h (n=2) or 72h (n=6) of rhIFN-γ treatment in relation to the norm population (200mg/dl glucose, 0ng/ml rhIFN-γ 0mM SB); **(F)** Percentages of the cell count of living H520 cells stained with trypan blue solution after 48h (n=2) or 72h (n=6) of rhIFN-γ and SB treatment in relation to the norm population (200mg/dl glucose, 25ng/ml rhIFN-γ 0mM SB; **(G)** Geometric mean of CD119 expression on A549 cell surface after 72h cell culture, with 25ng/ml rhIFN-γ and additional 0, 1 and 5mM SB treatment and analyzed by flow cytometry, (n=6); **(H)** Geometric mean of CD119 expression on H520 cell surface after 72h cell culture (n=6). (*P < 0.05; **P < 0.01; ***P < 0.001; ****P < 0.0001). One-way ANOVA test was used for figure **(C, D, F)**, Kruskal-Wallis test was used for figure **(A, E, G, H)** All data are presented as mean values ± SEM. Parts of the ﬁgure were drawn by using pictures from Servier Medical Art and is licensed under a Creative Commons Attribution 3.0 Unported License (https://creativecommons.org/licenses/by/3.0/).

In order to assess the effect of SB on the expression of IFN-γ-R1, a receptor involved in apoptosis and also in immune defense of CD8+ T cells, which produce IFN-γ to fight against cancer cells (15), we performed a staining for CD119 (IFN-γ-R1) on A549 cells treated with SB for 72h. Our findings revealed a significant increase in IFN-γ-R1expression with 5mM SB treatment in both glucose concentrations compared to cells treated with 0mM SB (**Figure 2A**). In parallel, we investigated the mRNA expression of several genes after 48h cell culture and SB treatment (**Supplementary Figure S1A**). The mRNA levels of the *IFNGR1*-gene tended to increase in a similar manner (**Supplementary Figure S1B**).

The observed increase of the IFN-γ-R1 in A549 after SB treatment (**Figure 2A**) led us to the investigation of the combined treatment with 25ng/ml recombinant human (rh)IFN-γ and 0, 1, 5 mM SB (**Figure 2B**). RhIFN-γ alone lead to a statistically significant reduction of viable cells after 48h (both glucose conditions) and 72h (only in glucose containing condition), whereas in glucose deprived condition both groups (control and rhIFN-γ treated) were at nearly the same level after 72h normalized on the 200mg/dl glucose 0mM SB cell count (**Figure 2C**), which is altogether showing that rhIFN-γ treatment itself, also leads to a cell viability reduction. Next, we wanted to see if the combined treatment of rhIFN-γ with SB leads to a reinforced reduction of tumor cells. The percentage of viable cells (normalized to rhIFN-γ + 0mM SB in 200mg/dl glucose) significantly decreased after 48h and 72h, exposed to rhIFN-γ and SB. This decrease was most pronounced when treated with rhIFN-γ and 5mM SB, regardless of the glucose concentration (**Figure 2D**). Next, we evaluated the rhIFN-γ treatment on H520. Here the viable cells tended to decrease in the presence of glucose with 5mM SB after 48h, whereas the same condition after 72h tended to increase, indicating that rhIFN-γ treatment alone may not have an effect on cell viability of H520 (**Figure 2E**). Combined treatment of rhIFN-γ and SB in the presence of glucose resulted in the previously observed trend of a decrease in viable cells at 48h and a significant decrease at 72h with SB treatment. (**Figure 2F**).

Next, we had a look, if the rhIFN-γ treatment had an impact on the IFN-γ-R1 cell surface expression in A549. Similar to the first set of experiments, we observed a significant upregulation of the cell surface expression of IFN-γ-R1 (CD119) equally in both glucose concentrations after treatment with 5mM SB (**Figure 2G**). The mRNA levels of *IFNGR1* showed the same tendency (**Supplementary Figure S1C**). The expression of IFN-γ-R1 on H520 cell surface does not show any change in the absence of glucose, whereas in the presence of glucose, a significant upregulation in the cell surface expression was observed after 5mM SB treatment. This effect was, in contrast to the A549, not observed after the application of rhIFN-γ in combination with SB treatment (**Figure 2H**).

### 3.3 SB induced apoptosis in lung cancer cell lines and NSCLC patient derived epithelial cells

**Figure 3:**
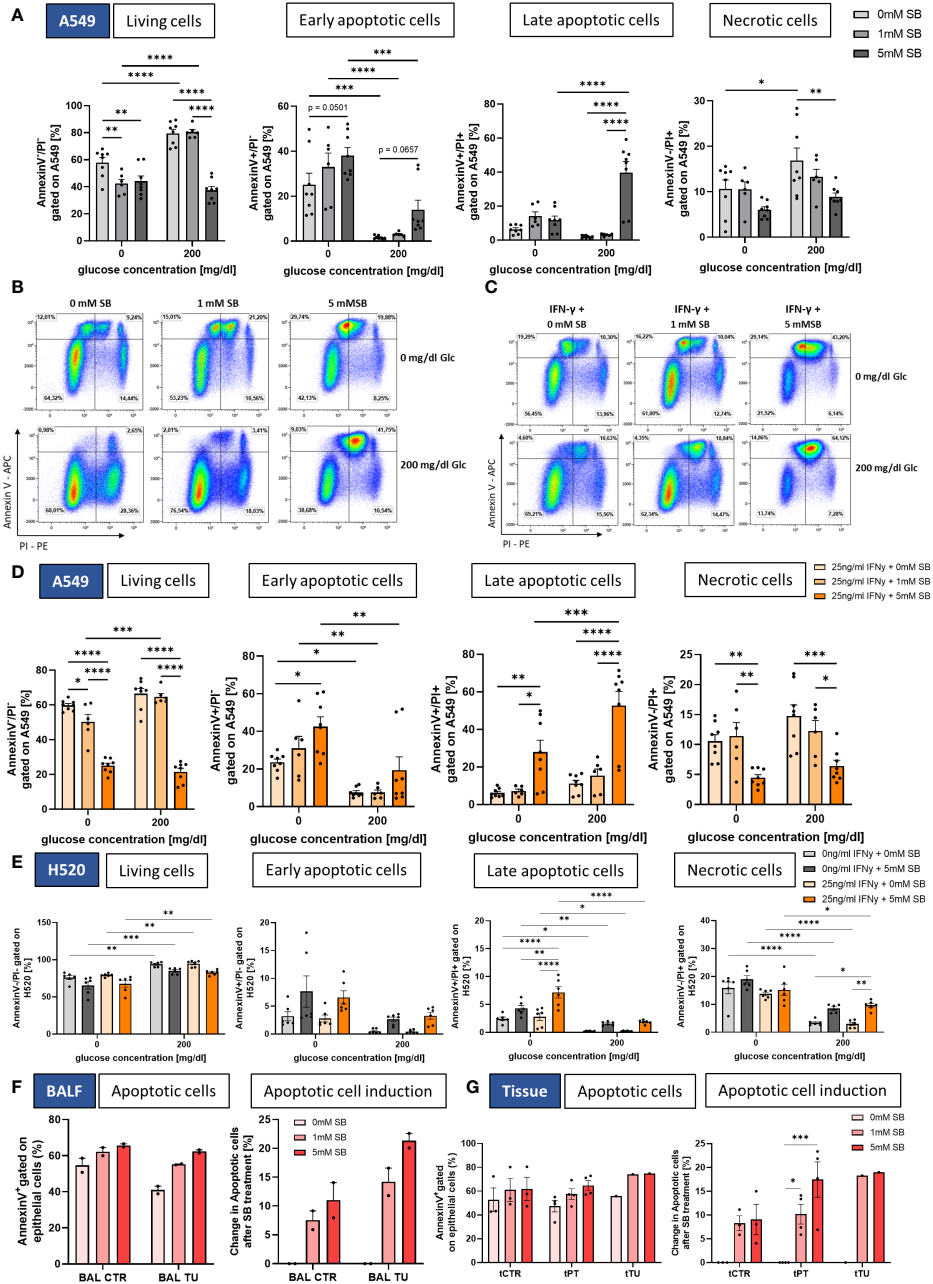
SB induced apoptosis in lung cancer cell lines and NSCLC-patient-derived cells. **(A)** Percentages of Annexin V/PI FACS analysis of A549 cells: living cells (Annexin V^-^ PI^-^), early apoptotic cells (Annexin V^+^ PI^-^), late apoptotic cells (Annexin V^+^ PI^+^) and necrotic cells (Annexin V^-^ PI^+^), (n=8); **(B)** Dotplots of Annexin V/PI FACS analysis of A549 cells after treatment with SB; **(C)** Dotplots of Annexin V/PI FACS analysis of A549 cells after treatment with rhIFN-γ and SB; **(D)** Percentages of Annexin V/PI FACS analysis of living cells (Annexin V^-^ PI^-^), early apoptotic cells (Annexin V^+^ PI^-^), late apoptotic cells (Annexin V^+^ PI^+^) and necrotic cells (Annexin V^-^ PI^+^), (n=8); **(E)** Percentages of Annexin V/PI FACS analysis of H520 cells: living cells (Annexin V^-^ PI^-^), early apoptotic cells (Annexin V^+^ PI^-^), late apoptotic cells (Annexin V^+^ PI^+^) and necrotic cells (Annexin V^-^ PI^+^), (n=6); **(F)** Annexin V/PI FACS analysis of patient BALF derived epithelial cells, apoptotic cells (Annexin V^+^) and induction of apoptosis based on each patient with 0mM SB for control and tumor, (n=2); **(G)** Annexin V/PI FACS analysis of patient tissue derived epithelial cells, apoptotic cells (Annexin V^+^) and induction of apoptosis based on each patient with 0mM SB for control and tumor, (n^control^=3, n^peritumoral^=4, n^tumor^=1); (*P < 0.05; **P < 0.01; ***P < 0.001; ****P < 0.0001). Two-way_ANOVA test was used for figure **(A, D, E)**, one-way ANOVA test was used for figure (**F, G** right panel) Kruskal-Wallis test was used for figure (**G** left panel). All data are presented as mean values ± SEM.

Furthermore, to investigate if the cell count difference is due to cancer cell death via early or late apoptosis (programmed cell death) or necrosis, we stained the cells after 72h with Annexin V and PI and analyzed them by FACS (**Figures 3A, B**). With 5mM SB concentration without or in the presence of 200 mg/dl glucose, we discovered a significant decrease of living cells (Annexin V^-^ PI^-^) compared to SB-untreated cells, confirming the results of the cell count in **Figure 1C** (**Figure 3A**, left panel). Upon examining early apoptosis (Annexin V^+^ PI^-^), we detected an upward trend in early apoptotic cells with 5mM SB treatment in both glucose conditions. In the absence of glucose, there was already a significant higher occurrence of early apoptotic cells compared to the 200mg/dl glucose condition among the SB untreated cells, and this increased further upon the addition of 1 or 5mM SB (**Figure 3A**, left middle panel). The late apoptotic cells (Annexin V^+^ PI^+^) exhibited a significant increase upon treatment with 5mM SB in the presence of glucose, whereas 0 or 1mM SB had no impact on late apoptosis. Conversely under glucose-deprived conditions, there was no observable alteration in late apoptosis (**Figure 3A**, right middle panel). In terms of necrotic cells (Annexin V^-^ PI^+^), SB treatment displayed an opposing effect, resulting in reduced percentages with higher SB concentrations, significant in the presence of glucose. However, this not significant trend was also observed in the absence of glucose. Here, the difference between both glucose concentrations was smaller compared to the variations observed in early and late apoptosis (**Figure 3A**, right panel). Apparently, we did not detect a significant increase in the expression of apoptosis related genes, but we observed an increased trend in the mRNA levels of Bax, Casp-9 and Bcl-2, and a slightly decreasing trend for Casp-8 (**Supplementary Figures S1D-G**).

As shown in **Figures 3C, D**, the combined treatment of rhIFN-γ revealed a significant decrease in the percentage of living cells with higher SB concentrations in both glucose concentrations with Annexin V-PI-staining (**Figure 3D**, left panel) and a significant increase of early apoptotic cells (Annexin V^+^ PI^-^) only in the absence of glucose with 5mM SB, but we detected an upward trend of early apoptosis with 200mg/dl glucose. As observed before without rhIFN-γ, there was already a higher occurrence of early apoptotic cells in the absence of glucose among the SB untreated cells as well as for the SB treated (**Figure 3D**, left middle panel).

The late apoptotic cells (Annexin V^+^ PI^+^) exhibited a significant increase in both glucose conditions upon treatment with 5mM SB, whereas 0 or 1mM SB had no high impact on late apoptosis. The 200mg/dl glucose and rhINF-γ + 5mM SB column is significantly higher than the one in absence of glucose (**Figure 3D**, right middle panel). The induction of late apoptosis tended to be stronger with rhIFN-γ treatment as compared to the condition without rhIFN-γ, where the late apoptotic cells tended to increase less strong (compare **Figure 3A**, right middle panel). In terms of necrotic cells (Annexin V^-^ PI^+^), SB treatment displayed an opposing effect in both glucose concentrations, resulting in reduced percentages with 5mM SB concentrations (**Figure 3D**, right panel).

A significant reduction of viable cells upon 5mM SB and the combination treatment with rhIFNγ in H520 was not observed, but the amount of viable cells in the absence of glucose was significantly lower compared to the viable cells in the presence of glucose (**Figure 3E**, left panel). The early apoptotic cells exhibited a not significant tendency to increase after administration of 5mM SB in both glucose concentrations (**Figure 3E**, left middle panel), whereas a trend of induction in late apoptotic cells was observed in 5mM ± rhIFN-γ treated cells in both glucose concentration, but compared to the A549 on a lower level. A notable increase in late apoptosis with rhIFN-γ and 5mM SB treatment was observed in the absence of glucose, along with a significantly higher percentage when compared to the effects of 5mM SB treatment alone, further highlighting the synergistic impact of rhIFN-γ and 5mM SB treatment (**Figure 3E**, right middle panel). Additionally, a trend of increasing necrotic cells was evident in the presence of glucose and significant with rhIFN-γ treatment (**Figure 3E**, right panel). In the late apoptotic cells and the necrotic cells, the absence of glucose resulted in a significantly higher amount of apoptotic and necrotic cells in all treatment groups (**Figure 3E** right middle and right panel). Altogether a similar trend of 5mM SB± rhIFN-γ treatment in H520 compared to A549 was evident, but the late apoptosis induction in 200mg/dl glucose was more than 20-fold higher in A549 (compared to **Figure 3A**, right middle panel) and the necrotic cells were induced in H520, whereas in the A549 a decreasing trend was visible.

For the NSCLC patient-derived epithelial cells we also wanted to see if SB is able to induce apoptosis. Here, the apoptotic cells showed a tendency to increase and the apoptotic cell induction (normalized on the 0mM SB control) showed a clear increase of apoptotic cells in the BALF-derived cells. The highest tendency of induction of apoptotic cells (Annexin V^+^) was observed with 5mM SB treatment and was even higher in the bronchial epithelial cells from the tumoral region compared to the control region (**Figure 3F**). In the tissue-derived cells the highest percentage of apoptotic cells (Annexin V^+^) was observed with 5mM SB treatment, with a tendency for an even higher percentage in the peritumoral and tumoral region of the alveolar epithelial cells. The apoptotic cell induction in PT (normalized on the 0mM SB control) displayed a significant increase in apoptosis compared to the 0mM SB treated cells. Additional evident here is the trend of a higher induction of PT and TU compared to the healthy control tissue derived cells (**Figure 3G**).

### 3.4 SB treatment led to cell cycle arrest through upregulation of CDK-inhibitors, downregulation of CDKs and induced the expression of the cancer stem cell marker CD90 in A549 cells

**Figure 4:**
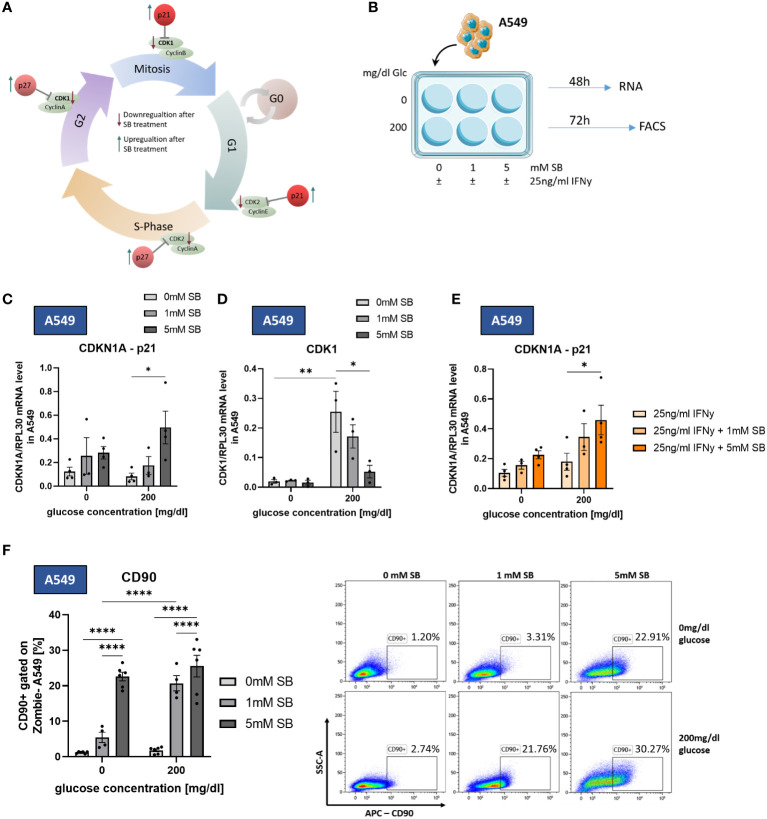
SB induced cell cycle arrest trough upregulation of p21 and downregulation of CDK1. CD90 surface expression increased upon SB treatment. **(A)** Schematic representation of cell cycle regulating proteins influenced by SB. SB leads to a lower gene expression of the cyclin-dependent kinases (CDKs), which are required for the normal cell cycle (green). The counterpart of the CDKs are kinase inhibitors, like p21, p27 and p16 (red). These inhibitors stopping the cell cycle at each checkpoint and therefore the proliferation of the cell; **(B)** qPCR analysis of relative CDKN1A(p21)/RPL30 mRNA expression in SB treated A549, (n=4); **(C)** qPCR analysis of relative CDK1/RPL30 mRNA expression in SB treated A549, (n=4); **(D)** qPCR analysis of relative CDKN1A/RPL30 mRNA expression in SB treated A549, (n=4); **(E)** A549 cells treated with SB were stained with anti-CD90 antibody and analyzed with flow cytometry, (n=6); (*P < 0.05; **P < 0.01; ****P < 0.0001). One-way ANOVA test was used for figure **(D, F)**, Kruskal-Wallis test was used for figure **(C, E)**. All data are presented as mean values ± SEM.

To further investigate how SB leads to a lower number of living cells, we studied possible changes in cell cycle regulating proteins, such as the inhibitors p21, p27 and the pro proliferating cyclin-dependent-kinases 1 (CDK1), cyclin-dependent-kinase 2 (CDK2) (cell cycle schematic illustrated in **Figure 4A**). For mRNA analysis A549 were cultured with SB (0, 1. 5 mM) and with or without glucose and 25ng/ml rhIFN-γ for 48h (**Figure 4B**). The mRNA levels of CDKN1A (p21) showed a significant increase following 5mM SB treatment in 200mg/dl glucose medium (**Figure 4C**). In accordance with this result, the mRNA levels of CDK1 were significantly decreased following 5 mM SB in comparison with untreated cell populations in 200mg/dl glucose medium. Under glucose-depriving conditions the CDK1 mRNA remained at a consistently low level (**Figure 4D**). The mRNA levels of CDKN1B (p27) tended to be increased upon SB treatment, in parallel with a decreasing trend of CDK2 (**Supplementary Figures S1H, I**). With combined treatment of rhIFN-γ and SB in the presence of glucose, the mRNA levels of CDKN1A (p21) significantly increase in a SB concentrations dependent manner, similar to the experiments without rhIFN-γ (**Figure 4E**). The expression of the CDKN1B (p27) gene showed a similar increasing tendency owing to SB treatment but this effect, was not statistically significant in our data (**Supplementary Figure S1J**).

To better characterize the phenotype of the surviving A549 cancer cells, we stained the cell surface of A549 cultured for 72h in different glucose conditions and SB (0, 1, 5mM) treatment (**Figure 4B**) with an antibody against CD90, a marker for cancer stem cells (CSC) in A549 (11), in flow cytometry. Upon SB treatment, we found significantly increased proportions of CD90+ A549 in both glucose conditions after 5mM SB treatment. A visible difference between the two glucose concentrations is evident at 1mM SB treatment. Specifically, at 1mM SB and 0mg/dl glucose medium the percentage of CD90+ A549 is significantly lower compared to the same SB concentration in the 200mg/dl glucose medium (**Figure 4F**).

### 3.5 SB reverses IFN-γ induced CD95-upregulation in A549 and H520 and reduced CD95 expression in NSCLC-patient-derived epithelial cells

**Figure 5:**
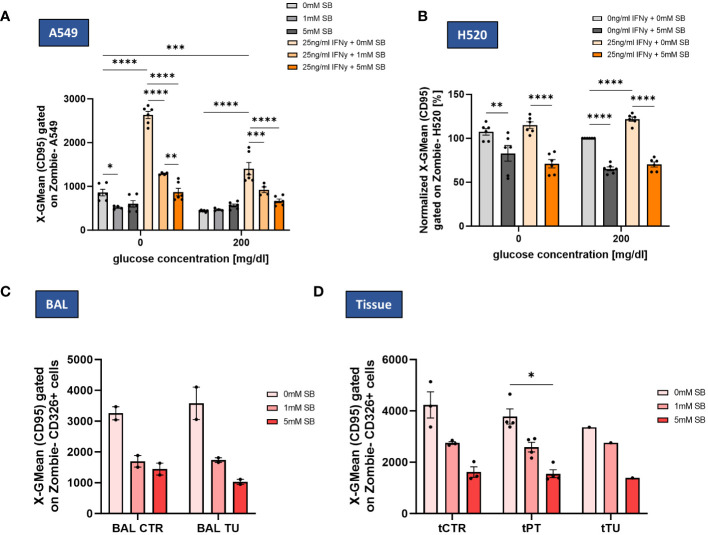
SB decreased IFN-γ induced CD95 cell surface expression in lung cancer cell lines and NSCLC patient-derived cells. **(A)** SB reversed IFN-γ induced CD95 upregulation, geometric mean of CD95 expression on A549 cell surface after 72h analyzed by flow cytometry, (n=6); **(B)** Geometric mean of CD95 expression on H520 cell surface after 72h of cell culture normalized to the control population (200mg/dl glucose, 0mM SB, 0ng/ml IFN-γ), (n=6); **(C)** Geometric mean of CD95 surface expressing cells gated on CD326+ Zombie- BALF epithelial cells in control and tumor regions analyzed by flow cytometry (n^control^=2; n^tumor^=2); **(D)** Geometric mean of CD95 surface expressing cells gated on CD326+ Zombie- tissue derived epithelial cells in control, peritumoral and tumor regions analyzed by flow cytometry, (n^control^=3, n^peritumoral^=4, n^tumor^=1); (*P < 0.05; **P < 0.01; ***P < 0.001, ****P<0.0001). One-way ANOVA test was used for figure **(A, B)**, Kruskal-Wallis test was used for figure **(C, D)** All data are presented as mean values ± SEM.

Activated effector γδT, Th1 and Tc1 CD8+ T cells can induce tumor cell death via cytokines or direct cell-cell contact by using cell surface molecule like Fas ligand (FasL). Fas and FasL are members of the tumor necrosis factor (TNF)-receptor and TNF family, respectively. The ligation of Fas (CD95) on the tumor cell with FasL, results in the activation of a caspase cascade that initiates apoptosis (16). To investigate by which mechanism SB is leading to a higher apoptotic rate, we studied the CD95 expression levels on the cell surface via flow cytometry. We found a decreasing effect of SB treatment on the surface expression of CD95 on A549 but only in the absence of glucose and treatment with 1mM SB, whereas in the presence of glucose no significant changes were evident (**Figure 5A**). After treatment with rhIFN-γ, the CD95 expression on the cell surface increased approximately 3 times (0mg/dl glucose) or 2 times (200mg/dl glucose) compared to the rhIFN-γ and SB non-treated cells (**Figure 5A**). After treatment with rhIFN-γ and 5mM SB the CD95-expression significantly decreased to a similar low geometric mean as without rhIFN-γ. (**Figure 5A**). In contrast to A549 cells, treating H520 cells with SB alone resulted in a significant reduction in CD95 cell surface expression in both glucose concentrations. Similarly to A549 cells, rhIFN-γ treatment also induced an increase in CD95 expression, which was significant in the presence of glucose. This increase was counteracted by the administration of 5mM SB bringing the expression levels back to a similar level as observed with SB treatment alone (**Figure 5B**).

In BALF-derived CTR and TU regions, the CD95 expression, tended to decrease with SB, but even more in the tumoral region (**Figure 5C**). In the tissue derived cells the expression of CD95 tended to decrease in all cell populations (CTR, PT and TU region). CD95 decreased in PT significantly after administration of 5mM SB (**Figure 5D**).

### 3.6 SB treatment reduced effector CD8+ T cells in PBMC especially in patients with lung cancer and increased CD4+ T cells independently from tumor or control patients

**Figure 6:**
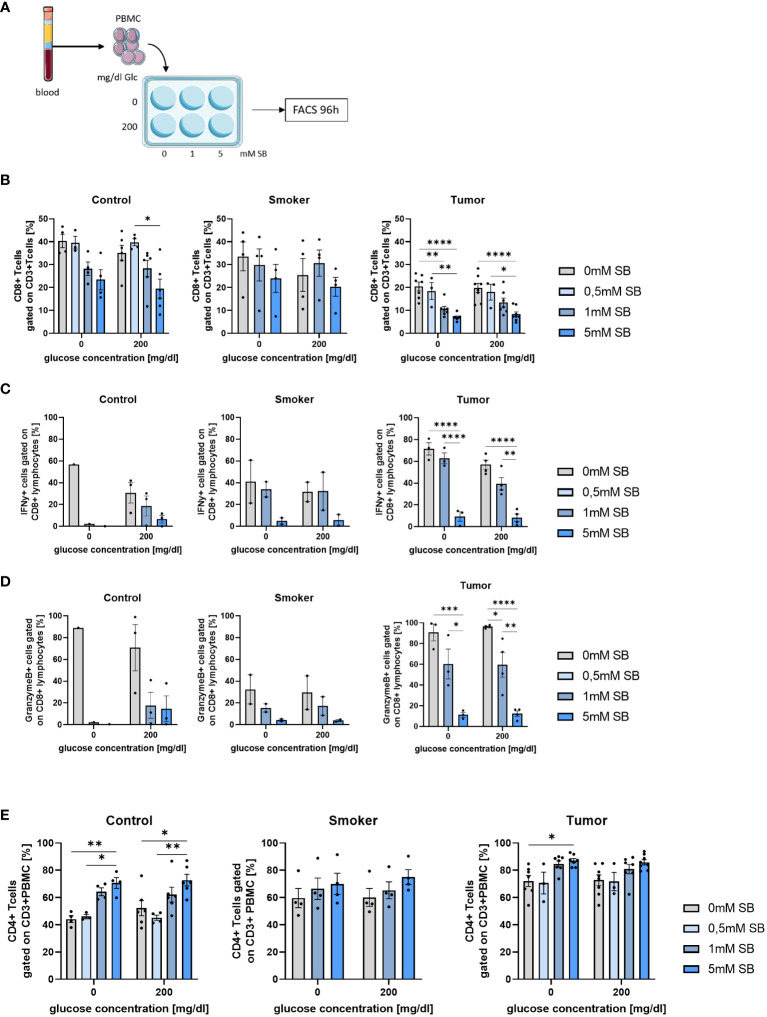
CD8+ T cells and their IFN-γ and Granzyme production decreased after 96h SB treatment *in vitro* with different glucose levels; Percentages of CD4+ T cells increased upon SB treatment in all populations **(A)** Schematic illustration of the experimental design. 5x10^5^ PBMC from human patients per well with 500µl medium were incubated for 96h with 200mg/dl glucose or without and SB concentrations increased from 0mM, 0.5mM over 1mM to 5mM SB; **(B)** Percentages of CD8+ T cells gated on CD3+ Zombie- lymphocytes in control, smoker and tumor patients, (n^control^=6; n^smoker^=4; n^tumor^=8); **(C)** Percentage of IFN-γ production in CD8+ T cells after treatment with 0, 1, 5mM SB and aCD3/28 stimulation for 96h analyzed by flow cytometry, (n^control^=3; n^smoker^=2; n^tumor^=4); **(D)** Percentages of granzymeB production in CD8+ T cells treated with 0,1, 5mM SB and aCD3/28 stimulation for 96h; analyzed by flow cytometry, (n^control^=3; n^smoker^=2; n^tumor^=4); **(E)** Percentages of CD4+ CD3+ T cells gated on lymphocytes, cultured for 96h as described in **Figure 4A**, analyzed by flow cytometry, (n^control^=6; n^smoker^=4; n^tumor^=8); (*P < 0.05; **P < 0.01; ***P < 0.001; ****P < 0.0001). One-way ANOVA test was used for figure (**B** middle and left panel, **C–E**) Kruskal-Wallis test was used for figure (**B** left panel). All data are presented as mean values ± SEM. Parts of the figure were drawn by using pictures from Servier Medical Art and is licensed under a Creative Commons Attribution 3.0 Unported License (https://creativecommons.org/licenses/by/3.0/).”.

To get an overview of the effect of SB on cancer, it is also important to take the immune system into account. For this purpose, PBMCs from healthy controls, smokers and NSCLC patients before lung surgery were cultured with or without glucose and increasing concentrations of SB for 96h (**Figure 6A**) and were analyzed by FACS with the gating strategies shown (**Supplementary Figure S2**). In all groups, SB tended to decrease the CD8+ T cell populations (**Figure 6B**). Low SB concentrations of 0.5mM SB did not show an effect on the percentages of CD8+ T cells. The amount of CD8+ T cells were highly variable in the smoker group. The tumor patients generally revealed lower percentages of CD8+ T cells after culture in comparison to the control and smoker cohort and decreased significantly upon treatment with 5mM SB. The glucose concentration did not seem to influence the percentages of CD8+ T cells (**Figure 6B**). Next, we had a look at the cytokine production in these CD8+ T cells via FACS. The IFN-γ-production decreased in all groups but especially in the tumor group, where we saw significant decreases in the 5mM SB treated group. Aside from the tumor patients, we, also noted a decreasing trend in the smoker and control cohort (**Figure 6C**). Similar effects were observed regarding the granzyme B production. Interestingly, the granzyme B production in the smokers was overall lower compared to the control and even compared to the tumor group. Again, an influence of glucose was not visible here as shown before (**Figure 6D**). We also had a look at the frequency of CD8+ effector memory T cells (TEM), which we gated as CD62L- CCR7- CD8+ CD3+ lymphocytes. In control and tumor patients the TEM tended to increase after 0.5mM SB treatment but decreased with higher concentrations to approximately 50% of the starting percentage at 0mM SB, which was, not statistically significant, (**Supplementary Figure S3A**).

In contrast to the CD8+ T cells the CD4+ T cells increased upon 1 or 5mM SB treatment, but not in the absence of SB or with 0.5mM SB (**Figure 6E**). This effect was most strongly seen in the control group, where we observed a significant increase with 5mM SB treatment regardless of the glucose concentration (**Figure 6E**, left panel). Additionally, the CD4+ T cell percentages in comparison of 0.5mM SB and 5mM SB also showed a clear difference as well in both glucose concentrations in control. In the tumor group (**Figure 6E**, right panel), a significant variation between 5mM SB and no SB was only seen without glucose, but the CD4+ T cells tended to increase also in the presence of glucose, whereas in the smoker group no statistically important changes where seen. The starting percentages at 0mM SB for the CD4+ T cells in the control patients were lower than in the smoker and tumor cohort (compare **Figure 6E**, left, middle and right panel). Additionally, as we did for the CD8+ T cells, we stained for the CD4+ TEM (CD62L- CCR7- CD4+ CD3+). In the control group as in the smoker no changes were visible, but in the tumor patients a slight decreasing trend with increasing SB concentrations was apparent, but not statistically significant (**Supplementary Figure S3B**).

### 3.7 Increased IFN-γ-R1 expressing NK cells induced by SB in PBMC from controls but not from lung cancer patients

**Figure 7:**
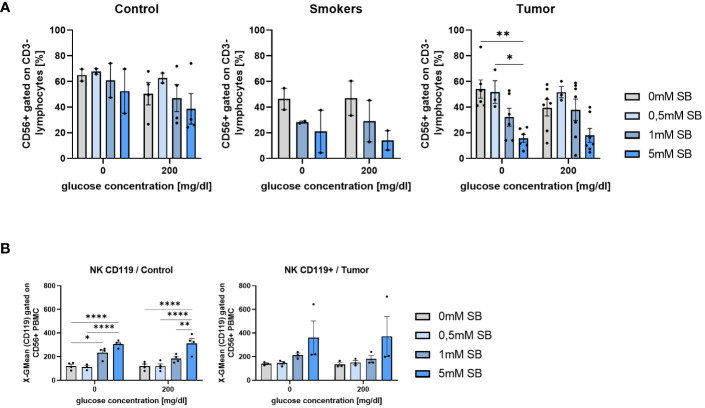
SB treatment led to decreased NK cell proportion in cultured PBMC from lung cancer patients **(A)** CD56+CD3- % NK cells of PBMC from control, smoker and lung tumor patients, cultured as described in **Figure 4A**, (n^control^=4; n^smoker^=2; n^tumor^=7); **(B)** Geometric mean of CD119 expression on NK cell (CD56+CD3-) surface after 96h, cultured as described in **Figure 4A**, analyzed by flow cytometry, (n^control^=4; n^tumor^=3); (*P < 0.05; **P < 0.01; ****P < 0.0001). One-way ANOVA test was used for figure (**A** right panel, **B** left panel) Kruskal-Wallis test was used for figure (**A** left panel, **B** right panel). All data are presented as mean values ± SEM.

To investigate the role of other tumor fighting immune cells besides CD8+ and CD4+ T cells, we had a look at the NK cells from the patient’s blood, gated as CD3- CD56+ cells. The NK cells tended to decrease in all groups after cell culture with increasing SB concentrations with a significant difference in the tumor group without glucose, although a similar tendency was observed with the 200mg/dl glucose (**Figure 7A**). At 200mg/dl glucose in the control group, as well as in the tumor group 0.5mM SB tended to increase the percentages of the NK cell population, whereas a higher SB concentration led to the same previously described decrease (**Figure 7A**). To further classify these NK cells, we also stained additionally for IFN-γ-R1 (CD119) on NK cells. Subsequent cell counts revealed a significant increase of CD119+ CD56+ CD3- cells following 5mM SB treatment compared to no SB. In the tumor group, the same effect might be present as we found a non-significant trend in the same direction (**Figure 7B**).

## 4 Discussion

An anti-inflammatory diet that is rich in dietary fiber, antioxidants, and an appropriate intake of protein, was suggested to play a preventive role in cancer development and may help to reduce the risk of both, the initiation and progression of lung cancer (17). In addition, such diet may support the regeneration of tissues and improve the nutritional status and cancer-related cachexia of individuals during the disease and after remission (17). Consumption of a diet high in saturated fatty acids, simple sugars, and highly processed foods, commonly known as the “Western” diet, can negatively alter the gut microbiome by causing an imbalance and promoting a pro-inflammatory environment (18, 19). The “Western” diet has been linked to an increase in opportunistic bacteria, as well as a decrease in the production of SCFAs (20). This type of diet may contributes to a chronic inflammation and the development of nutrition related diseases (19). However, recent findings indicate that the dysbiotic microbial changes caused by the “Western” diet can be reversed by supplementation with butyrate, as it has been demonstrated in an animal model (21). A healthy diet is also important for other organ systems like the lung and influences their health and disease via the gut-lung-axis (19), a bidirectional communication between the gut and the lungs, involving immune modulating components, such as microbial components, metabolites and signaling molecule (5).

In CRC patients altered gut microbiota were found, which was associated with altered metabolic profiles such as a reduced synthesis of SCFA (22), and further promoted colonic tumorigenesis. The modulation of the gut microbiota through a fiber rich diet and therefore higher SCFA production may have positive consequences on cancer progression and associated cachexia, as demonstrated in an animal model (23). Although SB was associated with many beneficial effects by promoting tumor suppression via HDAC inhibition, improved gut barrier function and reduced inflammation, there are also findings of opposite pro-tumoral effects (22).

Furthermore, in our and previous studies, high glucose concentrations were associated with pro-tumoral effects, which led to a better survival and proliferation of tumor cells (14). Glucose deprived SB untreated cells displayed a significantly lower cell number and higher early apoptosis rates compared to the glucose-cultured cells. Nevertheless, regarding the apoptosis rate SB effectively bypassed the higher apoptotic rate through induction of apoptosis in glucose-cultured cells, since 0mg/dl and 200mg/dl glucose in combination with 5 mM SB led to a comparable apoptosis rate. This fact may be helpful in tumor therapy, because a 0mg/dl glucose TME is physiologically not reachable. However, the cell count differences in untreated or 1mM SB treated cells may refer to a proliferation restriction of the glucose-deprived A549 and H520, which can be explained with the fact that cancer cells strongly rely on glucose in the TME, due to the Warburg effect (4). If the glucose deprivation may result in the observed overall low mRNA levels of CDK1 and the therefore low cell proliferation must be deeper investigated.

Apoptosis is a programmed cell death mechanism, which can remove unhealthy and damaged cells (24) and is mediated either by exogenous death receptors, like the TNFR-superfamily, or by endogenous mitochondria stress (25–27). Death receptors have the ability to activate Caspase 8 and therefore initiating a caspase downstream cascade leading to cell apoptosis (28). The intrinsic (endogenous) pathway involves the activation of pro-apoptotic proteins, like Bax, which neutralize Bcl-2, an anti-apoptotic protein. This leads to a change in the mitochondrial membrane potential and thus membrane damage. This triggers the release of pro-apoptotic factors in the cell and the activation of Caspase 9, which therefore activates Caspase 3 and leads to cell apoptosis (28).

In other cancer types like gastric cancer, SB has shown an induced apoptosis rate through upregulation of Caspase 9 and reduced Bcl-2 (29). A similar effect of SB was described, where the stimulation of endogenous apoptosis through SB treatment led to a reduced progress of cervical cancer (30). Based on our results, where a treatment with 5mM SB showed higher apoptosis rates, but decreasing necrosis rates compared to no treatment, we suggest that SB treatment may leads to apoptosis by potentially influencing an altered gene expression of apoptosis regulating proteins, which are required for the intrinsic mitochondrial pathway of apoptosis induction (28), attributable to the HDAC inhibitory effect of SB. The upregulating trend of Caspase 9 and Bax would confirm the intrinsic apoptosis induction. Although, the additionally upregulated Bcl-2 gene does not fit this hypothesis, further experiments on the RNA stability and the protein levels are needed. The effect of SB was stronger in the presence of glucose, because the lack of glucose alone already induced apoptosis and growth restriction in A549 cells and therefore concealed the effect of SB. The apoptosis rate observed in H520 after SB treatment was lower compared to the described findings in A549, with additionally increasing proportion of necrotic cells, which indicates that SB induced cell death in this cell line via both apoptosis and an apoptosis independent cell death form like necrosis. Additionally, the effect of SB in the H520 may depend more on a growth restriction than on apoptosis, since the cell count percentages are comparable to the A549, whereas the results of the apoptosis analysis indicate a more than 20-fold lower apoptosis rate induction through SB treatment. The patient-derived epithelial cells present apoptosis induction upon SB treatment, but independently of the exact origin of the cells, which may indicate that also the healthy cells of the control regions are unable to degrade SB and therefore also display an apoptosis induction as the peritumoral and tumoral derived cells.

In many previous studies HDAC inhibitors as SB have shown an influence on cell cycle regulating proteins, like p21, p27 (cyclin-CDK-inhibitors), CDK1, CDK2 (cyclin-dependent-kinases), and an induction of cell cycle arrest in G1/S and M/G2-phase in various cancer cells (13, 31, 32). A treatment with SB in prostate cancer cells resulted in an induced expression of p21 and p27 and effectively inhibited the proliferation of these cells (12) and moreover in H1299 lung carcinoma cells an upregulation of p21 and cell cycle arrest after SB treatment was found (33). In our study, 5mM SB treatment in A549 led to induced mRNA levels of p21 as well as reduced mRNA levels of one of the counterparts CDK1, indicating a cell cycle arrest in G1/S or G2/M-phase through HDAC inhibition activity of SB (33, 34).

Moreover, a major issue in tumor genesis are CSC, a specific cell subset within tumor cells exhibiting higher tumorigenicity and self-renewal capabilities (35). This is particularly important in various tumor types, as CSC have been considered as a contributor to resistance against chemo- and radiotherapy (36). These therapies target actively proliferating cells and therefore CSC can escape by going into cellular quiescence, a G0-resting state, without proliferation, where the cells can overcome vulnerabilities and maintain stemness (37). Several proteins like p21, p27 and p53 are upregulated determinants implicated in imparting quiescence (38).

Another mechanism of halting the cell cycle is mediated by elevated cell cycle inhibitor expression (p21, p16) due to DNA damage, stress or reactive oxygen species (ROS), which is called senescence (39). The cell stops proliferating to repair DNA damage or to undergo apoptosis. However, studies suggest that the cell cycle arrest associated with senescence might be reversible, enabling tumor cells to acquire a stem cell like state to overcome external stressors (40). Furthermore, there is an association between treating cells with 5mM SB with following HDAC6-inhibition, leading to the induction of senescent cells with a G2/M -phase cell cycle arrest in H1299 lung tumor cells through the upregulation of p21, as previously reported (33). The induction of senescence was also seen in CRC (22). This induction may align with our findings.

Additionally CD90 has been identified as a surface molecular marker for CSC in A549 (36). The observed increase in the expression of the stem cell marker expression CD90 in our experiments could potentially result from the reprogramming of SB-induced senescent cells into a stem-cell-like phenotype, which might help overcome and survive the external stress from SB treatment. A previous study highlighted an involvement of SCFA in blockade of cancer stem cell proliferation by altering the gene expression in the Wnt-ß-catenin pathway (41). Additional research is required to elucidate the precise mechanism and potential involvement of SB’s HDAC-inhibition in the upregulation of CD90 and the remaining effects of the increased stem cell population on cancer survival and progression.

Previously described data on CRC has shown that chronic exposure of CRC cells to SB may result in the selection of more aggressive clones as they upregulate genes involved in tumor angiogenesis and metastasis due to their ability to fully metabolize SB. The remaining cells may give rise to more aggressive cancer (42). More research is needed to see if NSCLC cells are able to gain the same metabolizing ability and therefore ensure their survival. This ability may limit the anti-cancer effects of SB.

Furthermore, SB could amplify the anti-tumor effect of the immune system as we found an upregulation of IFN-γ-R1 upon SB treatment in A549.

IFN-γ is a crucial proinflammatory cytokine that plays a pivotal role in protecting the host against tumor growth (43). It achieves this by directly inhibiting tumor cell growth, inducing cellular apoptosis, blocking neovascularization, and performing numerous immunomodulatory functions. For the initiation of IFN-γ signaling, the presence of IFN-γ R1 molecules on the cell surface is necessary. When IFN-γ binds to IFN-γ-R1, it triggers heterodimeric receptor binding, which in turn leads to autophosphorylation and activation of Janus-Kinase (JAK). Activated JAK phosphorylates the intracellular domain of the receptor and recruits STAT1 (Signal Transducer and Activator of Transcription 1) proteins (43). These proteins dimerize and translocate to the nucleus, where they induce the transcription of genes involved in apoptosis and cell cycle arrest via STAT1 dependent activation of CDK-inhibitors (44). Furthermore IFN-γ signaling can trigger, through the loss of NF-κB, necroptosis, which is a caspase-independent, regulated necrotic cell death. Important for that is the formation of a necrosome out of RIP1 and RIP3-kinase, which alters the metabolism of mitochondria resulting in their dysfunction and therefore leads to necroptosis. Many genes involved in this process are encoded by IFN-stimulated genes (45).

In this study we found an upregulation of IFN-γ-R1 upon SB treatment, which led us to the speculation that SB, as a HDAC-inhibitor, is involved in the molecular mechanisms that control the cell surface expression of IFN-γ-R1 and therefore facilitates IFN-γ-mediated apoptosis. Unlike the downstream signaling pathway, the molecular mechanisms by which IFN-γ-R1 expression on the cell surface is controlled, remain unclear (43). A549 and H520 treated with SB showed a comparable increase in the IFN-γ-R1 in the presence of glucose. IFN-γ itself had no effect on IFN-γ-R1 expression in A549 cells. In H520 cells, the presence of rhIFN-γ counteracts the SB-induced upregulation of IFN-γ-R1 observed in the absence of rhIFN-γ. Suggesting that this may be attributed to the increased internalization of the receptor through binding of rhIFN-γ (46). In other studies, the increase of IFN-γ-R1 on CRC lead to a higher chemotherapy sensitivity through apoptosis (47). Although, we did not investigate necroptosis and necroptosis-related genes in this study, we hypothesize that the main effect of the combination treatment of SB and IFN-γ refers to apoptotic cell death, because in A549 cells there is a high induction in apoptosis seen and no significant differences between the combined treatment or the one with SB alone.

Previous studies described that IFN-γ sensitizes tumor cells to rapidly undergo apoptosis by activating the Fas signaling pathway and enhances the cell death favoring effects of Fas on A549. Fas belongs to the TNF-R-family (Tumor necrosis factor receptor) and mediates cell death after ligand binding (Fas-L). The receptor monomers trimerize and recruit an intracellular death domain which leads to a downstream activation of the death inducing signaling complex (48).

In contrast to another study where no SB-related changes in the Fas expression were observed (17), SB treatment had a decreasing or the tendency of a decreasing effect on CD95 (Fas) in A549, H520 and patient derived epithelial cells from control, peritumoral and tumoral regions. After treatment with IFN-γ, the CD95-surface expression increased, but decreased with the combination treatment of IFN-γ and SB to the same levels as SB treatment alone, which suggests that SB reverses the IFN-γ-related increase in CD95 surface expression. This indicates that SB either leads to a decreased gene expression, an internalization of CD95 or a low expression of CD95 as an escape mechanism of the tumor cells, or the cells with a high CD95 expression died, leaving only the cells with a lower expression alive. In the latter case, SB induced apoptosis may be Fas-related. A previous study reported about immature glioblastoma cells which were resistant to Fas-induced apoptosis (48). This might be also the case in this study, explaining, why we observed a higher amount of A549 stem cells which expressed lower Fas than in the control, SB untreated, group.

Inducing apoptosis in cancer cells through cytokines, like SB, is one column in the fight against cancer, but the immune system of the patient is equally important. CD8+ T cells, also known as cytotoxic T-lymphocytes (CTL), are considered a key component of the body’s natural defense against cancer by recognizing and directly killing cancer cells. When activated, they can migrate to the site of tumor and release cytotoxic granules, which contain perforin, granzymes and IFN-γ and therefore induce apoptosis in the cancer cells. IFN-γ further enhances their cytotoxic activity and activates other immune cells to fight against the cancer (49). CD4+ T cells, can be pushed into an antitumor Th1 phenotype by IFN-γ signaling and therefore induce an upregulation of granzyme and IL-2 receptor on CD8+ T cells, helping these cells to their full cytotoxic potential (50). Earlier investigation showed the potential modulation of immune cells by microbial molecules (51). Previous studies reported a promotion of anti-tumor immunity of CD8+ T cells with an increased production of proinflammatory cytokines like IFN-γ, granzyme B and perforin and enhanced efficacy of chemotherapy through the production of metabolites, such as butyrate (10). However, in this study we observed the opposite effect in all patient-derived PBMC, independent from their origin, with decreased IFN-γ and granzyme B production after SB treatment, and the same effect was seen in NK cells. In this previously mentioned studies, some different cell culture methods were used, with purified CD8+ T cells and stimulation with Interleukin-2 (IL-2). Furthermore, the previously delineated pro-cytokine productive effect (10) manifested after 24 hours of treatment, and an extended treatment duration (96 hours) may negate this effect, leading to a prominence of cytotoxic effects of SB. The CD8+ T cell reduction and the inhibition of the cytokine production by SB may limit the beneficial SB induced proapoptotic effects on cancer cells through the suppression of CD8+ T cells, which may be insufficient in fighting against tumor cells without the required cytokine features. In fact, these cells are already reduced at the baseline in our cohort of patients with NSCLC. In contrast, clinical studies reported a correlation between the reduced abundance of SCFA-producing bacteria in PD-1 immunotherapy non-responders compared to the ones responding (52, 53) and recently, some results confirmed an association of SCFAs with the clinical responses to PD-1 blockade in cancer patients (54). However, which impact the decreasing CTL population has for the outcome of lung cancer patients, cannot be concluded from this study. Further *in vitro* and *in vivo* models are required to test, whether a combination treatment of SCFA with the current used immunotherapy options or chemotherapeutics can overcome the inhibition of the immune response inducing a sufficient immune system response and a beneficial outcome for the patients. Another limitation in this study is the utilization of PBMC instead of immune cells derived directly from the TME. Immune cells originating from the TME may experience immunosuppression and exhaustion due to tumor-derived communication signals, potentially leading to reduced cytokine production (55). Subsequent investigations could unveil whether SB exhibits a distinct anti-tumor impact on these CTLs and their cytokine production. In this study, PBMC were employed to discern potential differences between healthy control subjects and lung tumor patients.

Furthermore, we found that, SB treatment decreased NK cells but induced the IFN-γ-R1 surface expression on NK cells in control subjects.

NK cells can recognize and destroy cancer cells without the need for prior exposure or activation, by releasing cytotoxic granules and cytokines, such as IFN-γ and TNF-α, which directly induce apoptosis (cell death) in cancer cells. NK cells also have the ability to stimulate the adaptive immune response, through interactions with other immune cells such as dendritic cells and T cells, which is leading to a more robust and effective anti-tumor response. Overall, the ability of NK cells to target and kill cancer cells and stimulate the adaptive immune response make them an important player in cancer immunotherapy. In NSCLC an increased abundance of NK cells in the TME has been associated with a higher overall survival (50, 56). The decrease of peripheral NK cells in the PBMC population after SB treatment is considered detrimental for the general antitumor activity. Similar detrimental may be the low IFN-γ secretion of CD8+ T cells, because IFN-γ itself promotes the accumulation and cytotoxicity of NK cells via a self-regulatory feedback loop (57), in which the IFN-γ-R may play a crucial part. In our study we observed a significant increase in the surface expression of CD119 in NK cells of the control group after the SB treatment. The same trend was seen in the tumor group but without significance, which may be beneficial, especially if supported by a sufficient IFN-γ-concentration in the TME.

Regarding CD4+ T cells, we detected an increased percentage PBMC after SB treatment. CD4+ T cells play a crucial role in the immune response against cancer by activating and directing other immune cells, such as CD8+ T cells, NK cells, and macrophages, to attack cancer cells. They are involved in the formation of the immunological memory, which allows the immune system to recognize and eliminate cancer cells upon re-exposure. They can be classified in the helper T cell subtypes Th1, Th2, Th17, and regulatory T cells (Tregs) (58, 59). Depending on the cytokine milieu and immunological context, SCFA can support the development of either Th1 and Th17 effector cells or regulatory IL-10+ T cells. Characteristically Acetate (C2) and Propionate (C3) induced Th1 or Th17 associated genes, such as T-bet, and IFN-γ and IL-17A, IL17F, RORα, RORγt, respectively. SB showed a similar activity in this study at an effective concentration of 0.5mM through HDAC inhibition (11). SCFAs enhanced the mTOR pathway and STAT3 activation in T-cells and is therefore involved in expression of cytokines like IL-10, IL-17 and IFN-γ (11). The upregulation of the CD4+ T cells after SB treatment might be beneficial since there was no increased Treg population seen, as observed in some studies before, but the CD4+ cells have to be investigated more deeply to see, which subset of CD4+ T cells are upregulated through SB to understand if this could have a beneficial effect on the antitumor activity of the immune system. However, because the rise in CD4+ T cells appears to be approximately proportional to the decline in CD8+ T cells, there might be a connection, suggesting that the decrease in CD8+ T cells leads to the increase in CD4+ T cells, which would mitigate this effect on the CD4+ T cells.

Demonstrating the effects of SB in tumor cell lines of human origin, human primary epithelial cells and PBMC in highly controlled cell culture *in vitro* experiments formed the basis for further investigations in animal models. These are needed to further understand the effects of SB *in vivo* and also the interactions of the immune system with tumor cells and its associated TME. An animal model based on the results of this paper is ongoing in our workgroup. Furthermore, SB treatment *in vivo* is limited by its extremely short half-life, which is one reason for the use of SB releasing prodrugs, like tributyrin or AN-7 *in vivo*. Previous studies already investigated the use of pro-drugs, in combination with other therapeutic drugs *in vivo*, where the treatment led to a growth retardation of the tumors compared to the untreated group (60, 61). Additionally, the clinical use of SB might be limited by its physiological low availability (1-10µM (6)) in peripheral blood circulation. One solution for this problem might be a bacterial cancer therapy (BCT), where genetically modified butyrate producing bacteria are injected in the blood circulation and enriched in tumor tissues (62). Further investigations are needed before such therapies may be considered for clinical trials.

## 5 Conclusions

In this study, the anti-cancer effects of SB in NSCLC-cell lines and patient-derived epithelial cells have been demonstrated. The induction of apoptosis through upregulation of apoptosis-related genes and the observed alteration in the expression of cell cycle regulating proteins, notably the upregulation of p21 and the downregulation of CDK1, highlights the potential therapeutic impact of SB. The mechanism underlying the observed upregulation of IFN-γ-R1 post-SB treatment needs further investigations to elucidate its potential impact on therapy strategies. Additionally, the observed increase in CD4+ T cells following SB treatment might be beneficial for antitumor activity. However, it is essential to note the potential counteractive effects of SB therapy, as the emergence of a higher population of CSC after SB treatment coupled with a reduced expression of CD95 needs to be further investigated to understand their impact on cancer cell survival and disease progression. Moreover, the apoptosis induction of SB in patient-derived non-cancerous epithelial cells requires deeper exploration. Furthermore, the study shows suppressive effects of SB on parts of the immune system, particularly the decreased abundance of CD8+ T cells and their cytokine production, as well as the reduction in NK cells.

In conclusion, this study highlights the necessity for additional investigations to determine whether dietary (co-)therapy strategies, inclusive of dietary fibers, pre-, pro- and postbiotics or prodrug treatment leading to elevated SB concentrations, might manifest beneficial effects *in vivo*. Further research is vital to better comprehend the potential therapeutic implications and complexities of SB-based interventions in cancer therapy, considering both its anticancer effects and associated limitations on the immune system.

## Data availability statement

The original contributions presented in the study are included in the article/**Supplementary Material**. Further inquiries can be directed to the corresponding author.

## Ethics statement

The studies involving humans were approved by Local Ethic at the Uniklinikum Erlangen. The studies were conducted in accordance with the local legislation and institutional requirements. The participants provided their written informed consent to participate in this study.

## Author contributions

CT: Conceptualization, Data curation, Formal analysis, Investigation, Methodology, Project administration, Software, Validation, Visualization, Writing – original draft. PT: Formal analysis, Methodology, Writing – review & editing. KH: Formal analysis, Methodology, Writing – review & editing. ZY: Formal analysis, Methodology, Writing – review & editing. SK: Formal analysis, Methodology, Writing – review & editing. DT: Resources, Writing – review & editing. HS: Resources, Writing – review & editing. JS: Resources, Writing – review & editing. SF: Conceptualization, Funding acquisition, Project administration, Resources, Supervision, Writing – review & editing.
