# Supplementary material for: Short-chain fatty acids induced lung tumor cell death and increased peripheral blood CD4+ T cells in NSCLC and control patients ex vivo
Source: Front Immunol. 2024 Apr 8;15:1328263. doi: 10.3389/fimmu.2024.1328263 (PMC11033355; doi:10.3389/fimmu.2024.1328263)

Supplementary Material

Short-chain fatty acids induced lung tumor cell death and peripheral blood CD4+ T cells in NSCLC and control patients ex vivo

Carolin D. Thome[1], Patrick Tausche[1]†, Katja Hohenberger[1]†, Zuqin Yang[1], Susanne Krammer[1], Denis I. Trufa[2, 3, 4], Horia Sirbu[2, 3, 4], Joachim Schmidt[5] and Susetta Finotto[1, 3, 4*]

[1]Department of Molecular Pneumology, University Medical School Hospital Erlangen (UKER) Friedrich-Alexander-University (FAU) Erlangen-Nürnberg, Germany

[2]Department of Thoracic Surgery, University Medical School Hospital Erlangen (UKER), Friedrich-Alexander University of Erlangen-Nürnberg (FAU), Erlangen, Germany.

[3]Bavarian Cancer Research Center and Comprehensive Cancer Center Erlangen-EMN (CCC ER-EMN), Erlangen, Germany

[4]Comprehensive Cancer Center Erlangen-EMN (CCC ER EMN), Erlangen, Germany

[5]Department of Anesthesiology, University Medical School Hospital Erlangen (UKER), Friedrich-Alexander University of Erlangen-Nürnberg (FAU), Erlangen, Germany.

†These authors have contributed equally to this work

*** Corresponding Author:**

Prof. Dr. Dr. Susetta Finotto

Mail: susetta.finotto@uk-erlangen.de

http://www.molekulare-pneumologie.uk-erlangen.de

Department of Molecular Pneumology

Friedrich-Alexander-Universität Erlangen-Nürnberg

Hartmannstraße 14

91052 Erlangen, Germany

Supplementary Figure S1. Effect of SB on gene expression in treated A549 (A) Schematic illustration of the experimental design. $5x10^5$ A549 cells per well were incubated for 48 with 200mg/dl glucose or without and SB concentrations increased from 0mM over 1mM to 5mM SB with or without additionally 25ng/ml rhIFN-γ; **(B)** qPCR analysis of relative IFNGR1/RPL30 mRNA expression in SB treated A549, (n=4); **(C)** qPCR analysis of relative IFNGR1/RPL30 mRNA expression in SB and rhIFN-γ treated A549, (n=4); (**D**) qPCR analysis of relative Casp9/RPL30 mRNA expression in SB treated A549, (n=4); **(E)** qPCR analysis of relative Casp8/RPL30 mRNA expression in SB treated A54, (n=3); **(F)** qPCR analysis of relative Bcl-2/RPL30 mRNA expression in SB treated A549, (n=1); **(G)** qPCR analysis of relative Bax/RPL30 mRNA expression in SB treated A549, (n=1); (**H**) qPCR analysis of relative CDKN1B/RPL30 mRNA expression in SB treated A549, (n=4); **(I)** qPCR analysis of relative CDK2/RPL30 mRNA expression in SB treated A549, (n=4); **(J)** qPCR analysis of relative CDKN1B/RPL30 mRNA expression in SB treated A549 p(200mg/dl glucose: 0mM vs. 5mM SB)=0.1730 (n=4); One-way ANOVA test was used for figure (B-J). All data are presented as mean values ± SEM. Parts of the figure were drawn by using pictures from Servier Medical Art and is licensed under a Creative Commons Attribution 3.0 Unported License (https://creativecommons.org/licenses/by/3.0/).

Supplementary Figure S2. Gating strategies for different lymphocyte subpopulations in PBMC. Representative flow cytometry dot plots of PBMC from a control patient were generated to show the gating strategies. To identify distinct lymphocyte subsets, cells were prepared and stained with fluorochrome-labeled antibodies as outlined in the methodology section. Initially, doublets were excluded by gating on singlets using FSC-A against FSC-H. Next, lymphocytes were gated using FSC-A against SSC-A. To include only viable cells, staining with Zombie Aqua Dye was employed. For

NK cells, gating was performed on CD3- cells, followed by gating on CD56+ cells. T cells were identified by gating on CD3+ cells, and subsequently on CD4+ or CD8+ T cells. Further investigation of CD8+ T cells included assessing the expression of granzyme B or IFN-γ. Data sets were analyzed using Kaluza Flow Cytometry Software v2.1 (Beckman Coulter, Inc.).

Supplementary Figure S3. CD8+ TEM, CD4+ TEM (A) Percentages of CD62L-CCR7- T cells gated on CD8+ CD3+ Zombie- lymphocytes in control, smoker and tumor patients analyzed by flow cytometry ($n^{control}$=4; n^{smoker}=4; n^{tumor}=7); **(B)** Percentages of CD62L-CCR7- T cells gated on CD4+ CD3+ Zombie- lymphocytes in control, smoker and tumor patients analyzed by flow cytometry ($n^{control}$=4; n^{smoker}=4; n^{tumor}=7). One-way ANOVA test was used for figure (A right panel, B) Kruskal-Wallis test was used for figure (A left and middle panel). All data are presented as mean values ± SEM.

Supplementary Table S1: List of cell lines

**Experimental models: Cell lines**
Human A549 LUAD
Human H520
Patient derived cells

Supplementary Table S2: List of cell culture supplements

**Reagent**	**Source**	**Identifier**
rhIFN- γ	Immunotools	Cat#11343534
Sodium Butyrate	Sigma Aldrich	Cat#B5887-1G

Human aCD3	BD Pharmigen	Cat#555329
Human aCD28	BD Pharmingen	Cat#555725
PMA	Sigma Aldrich	Cat#524400
Ionomycin	Sigma	Cat#I3909-1ML
Golgistop	BD Pharmingen	Cat#51-2092KZ

Supplementary Table S3: Clinical Data of the Cohort analyzed in this study

**Patient-Code**	**Histological classification**	**Grading**	**TNM-stadium**	**Age**	**Gender**
MP-174	LUAD-LUSC	G3	IA2	74	Female
MP-175	LUAD	G2	IB	64	Male
MP-176	LUSC	G3	IA3	73	Male
MP-177	LUAD	G3	IA3	67	Female
MP-178	LUAD	G3	IA2	67	Male
MP-179	LUSC	G3	IA3	83	Male
MP-180	LUSC	G3	IIIB	65	Male
MP-181	LUSC	G3	IIB	81	Male
MP-182	LUAD	G1	IIIA	55	Female
iK1	n.a.	n.a.	n.a.	54	Female
iK11	n.a.	n.a.	n.a.	46	Male
iK12	n.a.	n.a.	n.a.	24	Male

iK13	n.a.	n.a.	n.a.	34	Female
iK14	n.a.	n.a.	n.a.	56	Female
iK15	n.a.	n.a.	n.a.	44	Female
CN529	n.a.	n.a.	n.a.	27	male

Abbreviations: Histological Classification: LUAD= Lung Adenocarcinoma, LUSC= Lung Squamous cell carcinoma; Histopathological Grading: G1 = well differentiated, G2 = moderately differentiated, G3 = poorly differentiated; n.a.=not applicable

TNM: T: describes the size of primary tumor, N: describes presence or absence and numbers of metastasis in the nearby lymph nodes, M: describes presence or absence of distant metastasis

Supplementary Table S4: Cell culture media used for human experiments

**Medium**	**Ingredients**
RPMI 0 mg/dl glucose	500 ml Gibco™ RPMI 1640 Medium, Thermo Fisher Scientific, no glucose (cat# 11879020); 50 ml heat-inactivated fetal bovine serum (FCS), Sigma-Aldrich (cat# S0615); 2mM L-Glutamine, anprotec (cat# AC-AS-0001); 5 ml Penicillin-Streptomycin (Pen/Strep), anprotec (cat# AC-AB-0024)
RPMI 200 mg/dl glucose	500 ml Gibco™ RPMI 1640 Medium, Thermo Fisher Scientific (cat# 21875091); 50 ml heat-inactivated fetal bovine serum (FCS), Sigma-Aldrich (cat# S0615); 2mM L-Glutamine, anprotec (cat# AC-AS-0001); 5 ml Penicillin-Streptomycin (Pen/Strep), anprotec (cat# AC-AB-0024)
Pneumacult Ex Plus Basal Medium	490ml Pneumacult Ex Plus Basal Medium, Stemcells Technologies^{TM} (cat# 05041); 500µl Hydrocortisone stock, Stemcells Technologies^{TM} (cat#07925); 10ml 10xSupplements
	Pneumacult + Medium<br><br>10ml Pneumacult Ex Plus Basal medium (with Supplements and Hydrocortisone); 100µl Antibiotikum/Antimykotikum (Gibco cat#15240096); 50µl Gentamycin, Sigma Aldrich (cat#G1272-100ML)
	Pneumacult++ Medium

	10ml Pneumacult +; 500µl heat-inactivated fetal bovine serum (FCS), Sigma-Aldrich (cat# S0615); 200µl Sodium Bicarbonate, GibCo (cat#25080-060)

Supplementary Table S5: List of human Flow Cytometry antibodies

**Reagent**	**Source**	**Identifier**
Zombie Aqua Dye, BV510	BioLegend	Cat#423102
Human TruStain FcX™ (Fc Receptor Blocking Solution)	BioLegend	Cat#422302
Mouse monoclonal anti-human-CD119, PE	BD Biosciences	Cat#558937
Mouse monoclonal anti-human-CD326, APC	BioLegend	Cat#324207
Mouse monoclonal anti-human-CD95, APC-Fire750	BioLegend	Cat#305637
Mouse monoclonal anti-human-CD90, AF647	BioLegend	Cat#328115
Mouse monoclonal anti-human-CD3, APC-Fire-750	BioLegend	Cat#300470
Mouse monoclonal anti-human-CD3, PE Cy5.5	eBioscience	Cat#35-0036-41
Mouse monoclonal anti-human-CD4, APC	eBioscience	Cat#17-0048-41
Mouse monoclonal anti-human-CD8, APC-Cy7	BioLegend	Cat#344714
Mouse monoclonal anti-human-CD56, BV421	BD Bioscience	Cat#562752
Mouse monoclonal anti-human-CD62L, BV421	BD Bioscience	Cat#563862
Mouse monoclonal anti-human-CD197 (CCR7), Pe-Cy7	BioLegend	Cat#353225
Mouse monoclonal anti-human- IFN- γ, AF488	eBioscience	Cat#53-7319-71

Mouse monoclonal anti-human/mouse GranzymeB, PE	BioLegend	Cat#396405
AnnexinV	BD Pharmingen	Cat#550474
Propidium Iodide	BD Pharmingen	Cat#556463
AnnexinVPI-Binding Buffer	BD Pharmingen	Cat#556454

Supplementary Table S6: List of primers used for Quantitative Real-Time PCR

**Gene**	**Primer Sequence**
hRPL30	fw: 5' – CTGGTGTCCATCACTACAGTGG – 3'<br>rev: 5' – CCAGTCTGTTCTGGCATGCTTC – 3'
hIFNGR1	fw: 5' – GTAGCAGCATGGCTCTCCTCT – 3'<br>rev: 5' – ATTAGTTGGTGTAGGCACTGAGGAC – 3'
hCDKN1A	fw: 5' – CTGGGGATGTCCGTCAGAAC – 3'<br>rev: 5' – CATTAGCGCATCACAGTCGC – 3'
hCDK1	fw: 5' – ACTACAGGTCAAGTGGTAGC – 3'<br>rev: 5' – TCCCGAATTGCAGTACTAGG – 3'
hFASR	fw: 5' – TGAAGGACATGGCTTAGAAGTG – 3'<br>rev: 5' – GGTGCAAGGGTCACAGTGTT – 3'
hCDK2	fw: 5' –CTCTTCCCCTCATCAAGAGC– 3'<br>rev: 5' – GGCTAGTCCAAAGTCTGCTA – 3'
hCDkN1B	fw: 5' – CTAGAGGGCAAGTACGAGTG – 3'<br>rev: 5' – ATGCGTGTCCTCAGAGTTAG – 3'

hCasp9	fw: 5' – GTCCTACTCTACTTTCCCAGG– 3'<br>rev: 5' –CAAAGATGTCGTCCAGGGTC– 3'
hCasp8	fw: 5' – AAGCAAACCTCGGGGATACT – 3'<br>rev: 5' – GGGGCTTGATCTCAAAATGA – 3'
hBcl-2	fw: 5' –GGCCTTCTTTGAGTTCGGTG– 3'<br>rev: 5' –AGTCATCCACAGGGCGAT– 3'
hBax	fw: 5' – TTTCGTTCAGGGTTTCATCT – 3'<br>rev: 5' – CACTTGAAGGTTGCCGTCAGA – 3'

Supplementary Fig. S1

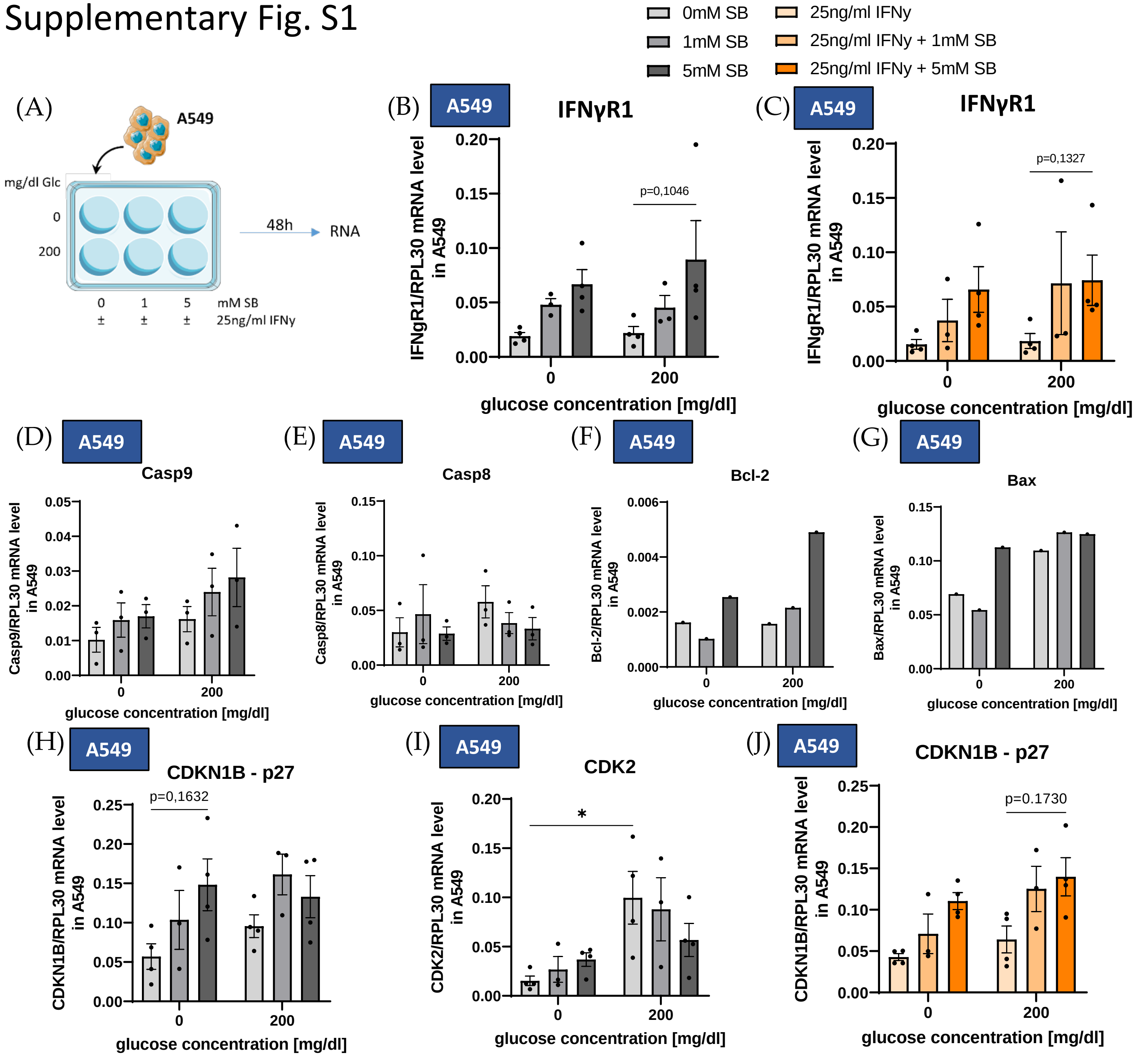

Supplementary Fig S2

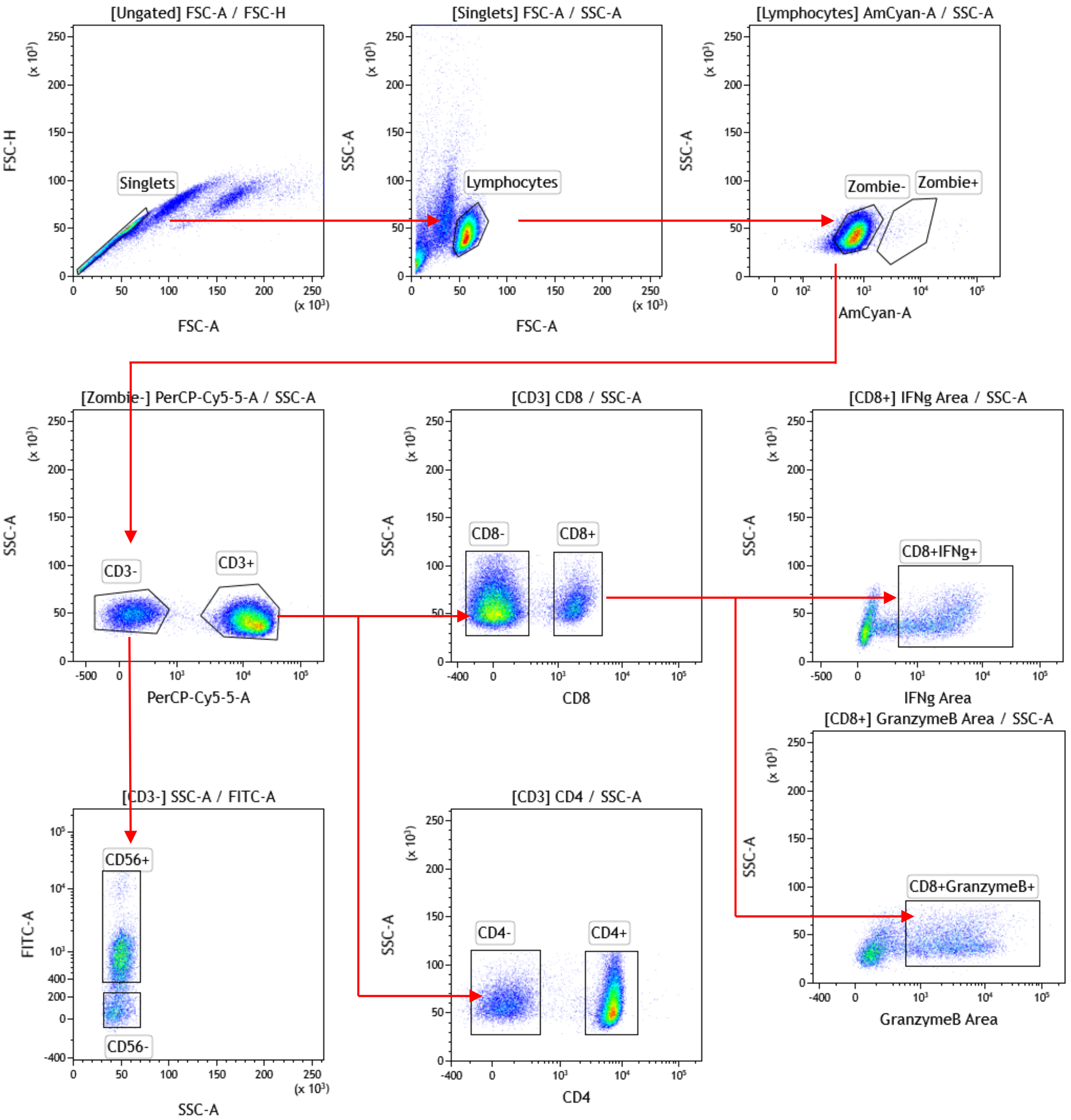

Supplementary Fig S3

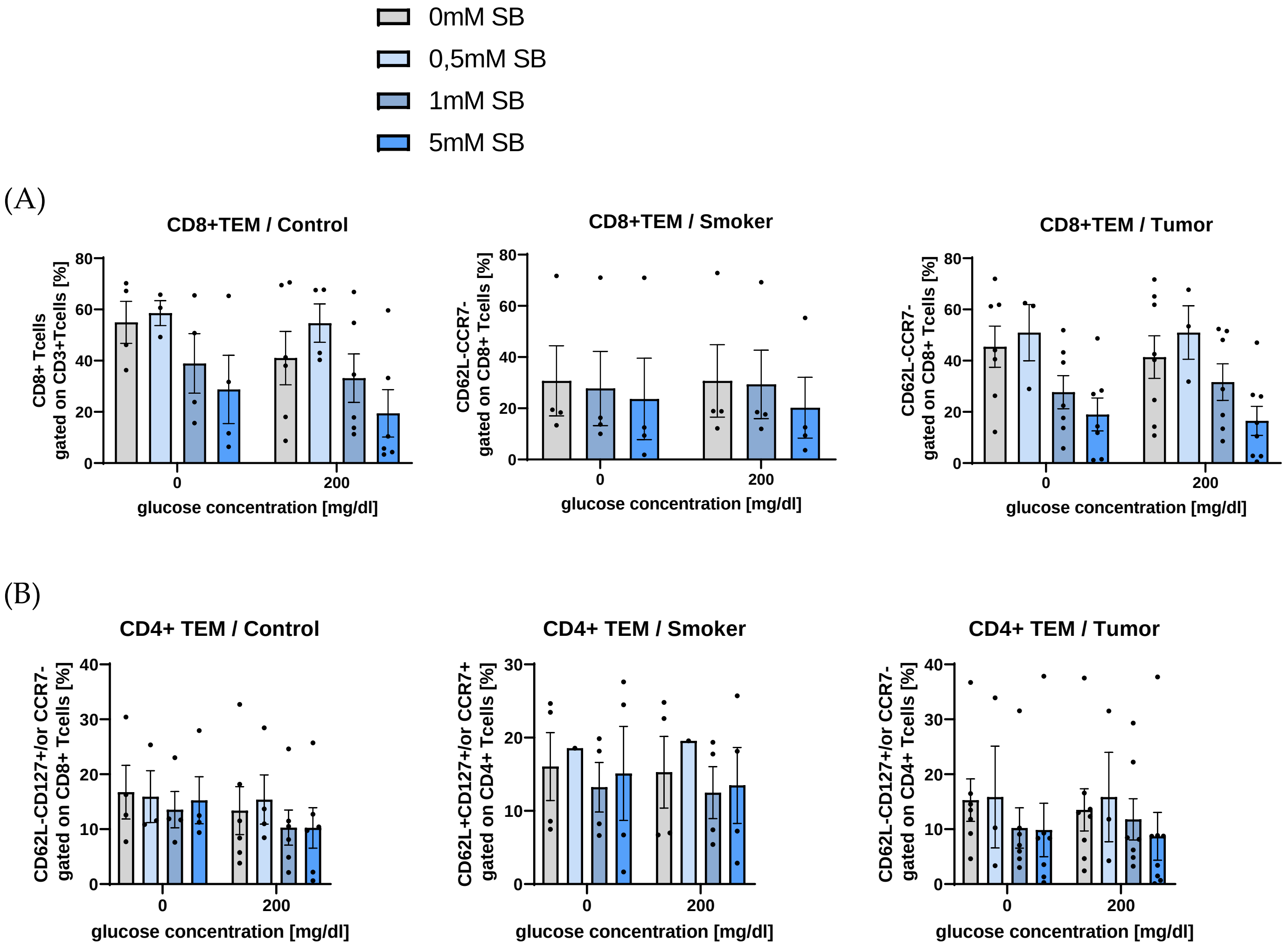

Supplement: Supplementary file 1 [file DataSheet_1.pdf]
